# The Association between Selenium and Other Micronutrients and Thyroid Cancer Incidence in the NIH-AARP Diet and Health Study

**DOI:** 10.1371/journal.pone.0110886

**Published:** 2014-10-20

**Authors:** Thomas J. O’Grady, Cari M. Kitahara, A. Gregory DiRienzo, Margaret A. Gates

**Affiliations:** 1 University at Albany, School of Public Health, Rensselaer, New York, United States of America; 2 Division of Cancer Epidemiology and Genetics, National Cancer Institute, National Institutes of Health, Rockville, Maryland, United States of America; University of Navarra, Spain

## Abstract

**Background:**

Selenium is an essential trace element that is important for thyroid hormone metabolism and has antioxidant properties which protect the thyroid gland from oxidative stress. The association of selenium, as well as intake of other micronutrients, with thyroid cancer is unclear.

**Methods:**

We evaluated associations of dietary selenium, beta-carotene, calcium, vitamin D, vitamin C, vitamin E, folate, magnesium, and zinc intake with thyroid cancer risk in the National Institutes of Health – American Association of Retired Persons Diet and Health Study, a large prospective cohort of 566,398 men and women aged 50–71 years in 1995–1996. Multivariable-adjusted Cox proportional hazards regression was used to examine associations between dietary intake of micronutrients, assessed using a food frequency questionnaire, and thyroid cancer cases, ascertained by linkage to state cancer registries and the National Death Index.

**Results:**

With the exception of vitamin C, which was associated with an increased risk of thyroid cancer (HR_Q5 vs Q1_, 1.34; 95% CI, 1.02–1.76; P_trend_, <0.01), we observed no evidence of an association between quintile of selenium (HR_Q5 vs Q1_, 1.23; 95% CI, 0.92–1.65; P_trend_, 0.26) or other micronutrient intake and thyroid cancer.

**Conclusion:**

Our study does not suggest strong evidence for an association between dietary intake of selenium or other micronutrients and thyroid cancer risk. More studies are needed to clarify the role of selenium and other micronutrients in thyroid carcinogenesis.

## Introduction

The past three decades have seen a rapid increase in thyroid cancer incidence in the United States (U.S.) and other countries [1]–[5]. Increased medical surveillance and diagnostic scrutiny are likely responsible for some, but not all, of this trend [6]. In addition to established risk factors such as ionizing radiation exposure and benign thyroid nodules [7], recent studies have focused on modifiable etiologic factors such as diet and obesity. Findings from these studies indicate that obesity and excessive weight gain during adulthood [8]–[10] and dietary nitrate and nitrite intake [11], [12] are associated with increased thyroid cancer risk while eating various fruits and vegetables [13]–[17], having adequate iodine intake and consuming fish [18], and a Polynesian-style diet [19] may be protective. Still, there is no consensus as to what dietary factors contribute to or inhibit thyroid carcinogenesis, as others have reported that a traditional Western diet [19], [20] and fruit and vegetable consumption [21] are unassociated with thyroid cancer risk.

While there has been research into dietary patterns as a whole, less work has been done to assess specific dietary constituents such as micronutrients and their impact on thyroid cancer risk. Additional research into the association between micronutrients and thyroid cancer may help further the understanding of the biological mechanisms involved in thyroid carcinogenesis. Selenium is an essential trace element that is found at higher concentrations in the thyroid gland than in other organs [22]. Selenium is important in the metabolism of thyroid hormones triiodothyronine (T3) and thyroxine (T4). Specifically, type I 5′-deiodinase is a selenium-containing protein which assists in activating naturally occurring T4 (which has little biological activity) into biologically active T3. Additionally, selenium has antioxidant properties which may help protect the thyroid gland from H_2_O_2_ and reactive oxygen species [23]–[26]. While selenium has been shown to have an inverse association with other cancers [27] this relationship has not been investigated for thyroid cancer. A recent meta-analysis of the association between supplemental micronutrients and thyroid cancer showed both that there is limited research on the topic of micronutrients and thyroid cancer and that the results of these prior studies are largely inconclusive [28].

We therefore examined the association between dietary intake of micronutrients and thyroid cancer risk in the U.S. National Institutes of Health-American Association of Retired Persons (NIH-AARP) Diet and Health Study, a large prospective cohort of 566,398 men and women ages 50–71 years at baseline. The primary micronutrient of interest was selenium because of its established importance for proper function of the thyroid gland, its potential antioxidant properties, and the possible inverse association between selenium and other cancers [23], [24], [27]–[29]. To our knowledge this is the first prospective evaluation of dietary selenium intake in relation to thyroid cancer risk. Additionally, we evaluated the associations between dietary intake of beta-carotene, calcium, folate, magnesium, vitamin C, vitamin D, vitamin E, and zinc with thyroid cancer risk to expand upon recent studies investigating supplemental intake of these micronutrients and thyroid cancer [28].

## Methods

### Study Population

The NIH-AARP Diet and Health study began in 1995–1996 with the mailing of an extensive baseline questionnaire to 3.5 million AARP members aged 50–71 years old and residing in six U.S. states (California, Florida, Louisiana, North Carolina, New Jersey, and Pennsylvania) or two U.S. metropolitan areas (Atlanta, Georgia and Detroit, Michigan). Information ascertained by the questionnaire included usual dietary intake over the past twelve months, use of individual and multivitamin supplements, alcohol intake, smoking history, height and weight at baseline, and other factors. Of the 566,398 individuals who were deemed to have satisfactorily completed the baseline questionnaire we excluded, in the following order, participants with proxy respondents (n = 15,760), those who reported poor health or end stage renal disease (n = 9,134), participants with a previous diagnosis of cancer other than non-melanoma skin cancer (n = 53,195), those with extreme or missing values for total energy intake (n = 4,279) and individuals with extreme values for daily selenium intake (n = 1,223). The remaining analytic cohort included 482,807 participants (287,944 men and 194,863 women). The NIH-AARP Diet and Health Study was approved by the Special Studies Institutional Review Board of the U.S. National Cancer Institute and the Institutional Review Boards from all participating institutions approved the use of these data.

### Cancer Ascertainment

Participants accrued time in the study from the date of baseline questionnaire completion to the date of any cancer other than non-melanoma skin cancer, the date when a person moved out of the registry ascertainment area, death, or December 31, 2006, whichever occurred first. Incident thyroid cancers (International Classification of Disease for Oncology, Third Edition (ICD-O-3), topography code C73) [30] were identified through December 31, 2006, via linkage of the NIH-AARP cohort membership to state cancer registries and the National Death Index [31]. The state cancer registries are certified by the North American Association of Central Cancer Registries as being at least 90% complete within two years of the close of the diagnosis year [32]. A validation study comparing linkage to state cancer registries with self-report and subsequent medical record conformation of incident cancers estimated that 90% of all cancer cases identified in the NIH-AARP Diet and Health Study were valid [31]. Subtypes of thyroid cancer were defined by ICD-O-3 morphology codes (papillary, 8050, 8052, 8130, 8260, 8340–8344) and (follicular, 8290, 8330–8332, 8335).

### Dietary Intake

The baseline questionnaire had a dietary component, which included questions about the frequency of dietary consumption during the past twelve months of 124 food items and the corresponding portion sizes of 100 of those food items. Intake frequency was recorded as one of ten categories ranging from “never” to “2+ times per day” for foods and “never” to “6+ times per day” for beverages. Additionally, each item included three possible portion size responses. The methods of Subar et al [33] along with national dietary data from the U.S. Department of Agriculture’s 1994–1996 Continuing Survey of Food Intake by Individuals (CSFII) [34] were used to construct the food items, portion sizes, nutrient database, and pyramid food servings database. A recipe file was used by the Pyramid Servings Database to disaggregate food mixtures into component ingredients and assign the components to food groups. The FFQs used by the NIH-AARP Diet and Health Study have been validated using two 24-hour recalls in a subset of the cohort [35]. The daily micronutrient consumption of an individual was determined by multiplying the frequency of consumption of each line item by its micronutrient content (determined from CSFII) and summing over all line items.

### Statistical Analysis

Cox proportional hazards regression [36] with person-years as the underlying time metric was used to estimate the cause-specific hazard ratios (HR) and corresponding 95% confidence intervals (CI) for thyroid cancer by quintiles of selenium and other micronutrient intake for thyroid cancer overall and the papillary and follicular subtypes. Quintiles of micronutrient intake were assessed first using the same cut points for men and women, resulting in an equal number of participants in each quintile when summed across men and women but not within sex. We then ran the analysis using separate, sex-specific, cut points for men and women. We assessed and verified, using cumulative sums of martingale residuals [37], that there was no violation of the proportional hazards assumption. We tested for linear trend by including the median value of each micronutrient category as a continuous variable in the model and assessing the significance of the Wald Chi-square p-value.

Micronutrient intake was adjusted for total energy intake using the nutrient residual method [38], which computes nutrient intake by first removing both calorie and micronutrient outliers, separately by sex, and then taking residuals from the regression model with total caloric intake as the independent variable and absolute nutrient intake as the dependent variable and adding a fixed constant (mean caloric intake by sex) of the study population.

Our minimal model was adjusted for age (continuous) and sex in the overall analysis and age in the sex specific analysis. Potential confounding variables were identified based on a review of the literature and previous studies on thyroid cancer using the NIH-AARP Diet and Health Study. Potential confounders were assessed using a backward elimination method in which we removed the least significant covariate in the model and assessed whether this changed the main exposure HR by more than 10%. We assessed for confounding by age (continuous), sex, body mass index (BMI; <18.5, 18.5–24.99, 25–29.99, >30), total calories (continuous), education (high school or less, some college, college or post graduate, unknown), physical activity (<1–2 times per week, 3–4 times per week, 5+ times per week, unknown), race (White, Black, other, unknown), smoking status (never, current, former, unknown), marital status (yes, no, unknown), alcohol intake (≤1, 2, 3, 4+ drinks/day), and micronutrient intake (continuous intake of vitamin C, vitamin E, beta-carotene, and folate). Effect modification was assessed using the likelihood ratio test comparing a model with the cross-product terms to one without.

Finally, we tested the assumption that the risk of thyroid cancer was log-linearly associated with selenium and other micronutrient intake by comparing the linear model with a non-parametric regression curve obtained with restricted cubic splines [39]. We used a stepwise selection process to identify the number and location of knots for each micronutrient analyzed. The likelihood ratio test was used to fit the restricted cubic splines. SAS software version 9.3 (SAS Institute, Cary, NC) was used for all statistical analyses. All reported p-values are based on two-sided tests and an alpha level of 0.05.

## Results

Over a total of 4,406,634 person-years of follow-up we identified 592 incident thyroid cancer cases (257 in men and 335 in women) of which 406 were of the papillary histologic subtype (164 in men and 242 in women) and 113 (57 in men and 56 in women) were of the follicular histologic subtype. Select characteristics of the study population are presented by quintile of dietary selenium intake (Table 1). Participants in the highest quintile of selenium intake were more likely to be married, never or former smokers, and college educated when compared to the lowest quintile of selenium intake. The mean and standard deviation for selenium intake was 94.0±42.9 mcg/day (other micronutrient mean and standard deviation intakes presented in Table 2). The five largest contributors to selenium intake were breads & rolls (13.9%), pasta (6.3%), tuna (4.8%), fish – not fried (4.1%), and eggs (4.0%). Contributors to vitamin C intake were orange & grapefruit juices (29.1%), oranges & tangelos (7.9%), broccoli (7.3%), other juices (5.1%), and grapefruit (4.5%). The dietary contributors of each micronutrient studied were similar for men and women. Table 2 provides a detailed summary of the major dietary contributors of each micronutrient in our study.

**Table 1:** Baseline characteristics of the NIH-AARP Diet and Health Study cohort by quintiles of residual adjusted selenium intake.

		Male N = 287,944					Female N = 194,683				
		Q1	Q2	Q3	Q4	Q5	Q1	Q2	Q3	Q4	Q5
	**N** * ** = **	**22,396**	**39,795**	**60,176**	**77,287**	**88,290**	**74,165**	**56,767**	**36,385**	**19,275**	**8,271**
	Number of thyroid cancer cases	19	25	49	68	96	119	106	66	29	15
	Number of papillary thyroid cancer cases	12	14	33	49	56	83	77	49	21	12
	Number of follicular thyroid cancer cases	4	8	8	14	23	21	14	14	5	2
	Energy adjusted Selenium quintile median (mcg/day Residual Method)	7.1	7.6	8	8.4	8.9	7.1	7.6	8	8.4	8.9
	Unadjusted Selenium quintile median (mcg/day)	47	68.4	86.6	108.6	150.1	47	68.4	86.6	108.6	150.1
**Parameter**	**Characteristics**	**Q1**	**Q2**	**Q3**	**Q4**	**Q5**	**Q1**	**Q2**	**Q3**	**Q4**	**Q5**
Age+		62.4 (5.4)	62.5 (5.3)	62.4 (5.3)	62.1 (5.3)	61.7 (5.4)	62.1 (5.4)	61.9 (5.4)	61.7 (5.4)	61.5 (5.4)	61.5 (5.4)
Race	White (%)	88.2	91.4	92.7	93.4	93.5	86.9	90.8	91.3	91.1	91.6
	Black (%)	5.6	3.4	2.5	2.2	2.1	7.8	4.4	4	3.9	3.8
	Other (%)	4.5	3.9	3.7	3.4	3.4	3.5	3.5	3.4	3.6	3.3
Currently Married (%)		79.6	83.9	85.6	86.4	85.7	41.7	45.9	46.9	46.4	43.9
BMI (kg/m^2^)		26.8 (4.2)	26.8 (4.1)	27.0 (4.1)	27.3 (4.2)	27.7 (4.5)	26.3 (5.7)	26.8 (5.9)	27.2 (6.1)	27.5 (6.3)	27.8 (6.4)
Smoking History	Never Smoker (%)	26.7	28.9	29.6	30	29.6	44.9	45	44	41.8	39.4
	Former Smoker (%)	53.6	55	56.2	56.6	57.8	35.1	38.5	40.1	42.7	45.1
	Current Smoker (%)	15.3	12	10.3	9.7	8.8	16.3	13.3	12.5	11.9	11.1
Education College Grad/PostGrad (%)		37.6	40.7	43.8	45.9	48.4	26.5	30.9	32.7	34.1	34.4
20 minutes Physical activity 5 or more times per week (%)		21.1	21.3	20.9	21.0	22.5	16.2	15.9	16.1	16.8	19.3
Post- menopause (% Yes)		n/a	n/a	n/a	n/a	n/a	94.5	93.9	93.8	93.5	93.2
Hormone Use (% Yes Ever)		n/a	n/a	n/a	n/a	n/a	51.3	55.2	55.2	54.5	51.5
Currently taking hormones (% yes)		n/a	n/a	n/a	n/a	n/a	42.2	46.1	46.2	45.5	43.2
Dietary Intakes (Vitamins Residually Adjusted)	Calories	2,226.0 (1,018.8)	1,995.4 (875.0)	1,935.1 (800.6)	1,944.1 (781.5)	2,064.9 (812.5)	1,568.6 (677.7)	1,522.9 (616.0)	1,561.0 (623.4)	1,623.0 (647.0)	1,702.1 (648.5)
	Vitamin C (mg/day)	9.7 (2.6)	9.6 (2.1)	9.5 (1.9)	9.4 (1.7)	9.2 (1.7)	9.5 (2.0)	9.2 (1.6)	9.1 (1.6)	9.0 (1.6)	8.9 (1.7)
	Vitamin D (mcg/day)	1.2 (0.8)	1.5 (0.8)	1.6 (0.7)	1.7 (0.7)	1.8 (0.6)	1.2 (0.8)	1.4 (0.7)	1.4 (0.6)	1.5 (0.6)	1.7 (0.7)
	Vitamin E (mg/day)	2.2 (0.5)	2.3 (0.4)	2.4 (0.4)	2.4 (0.4)	2.4 (0.3)	2.1 (0.4)	2.2 (0.3)	2.2 (0.3)	2.2 (0.3)	2.2 (0.3)
	Beta-carotene (mcg/day)	9.5 (1.2)	9.7 (1.1)	9.8 (1.0)	9.9 (1.0)	10.0 (1.0)	9.9 (1.1)	10.1 (1.0)	10.1 (1.0)	10.2 (1.0)	10.2 (1.1)
	Calcium (mg/day)	7.3 (0.6)	7.5 (0.5)	7.5 (0.5)	7.6 (0.5)	7.5 (0.4)	7.3 (0.5)	7.4 (0.5)	7.4 (0.5)	7.3 (0.4)	7.3 (0.4)
	Folate(mg/day)	12.8 (1.7)	13.3 (1.2)	13.4 (1.1)	13.6 (1.1)	13.8 (1.0)	12.5 (1.3)	12.8 (1.0)	12.9 (1.0)	13.0 (1.0)	13.1 (1.0)
	Magnesium (mg/day)	10.8 (1.0)	11.2 (0.7)	11.3 (0.7)	11.4 (0.6)	11.5 (0.6)	10.6 (0.8)	10.8 (0.6)	10.8 (0.6)	10.9 (0.6)	11.0 (0.6)
	Zinc (mg/day)	2.4 (0.4)	2.7 (0.4)	2.9 (0.3)	3.0 (0.3)	3.1 (0.3)	2.4 (0.4)	2.6 (0.3)	2.6 (0.3)	2.7 (0.3)	2.7 (0.3)

^*^The same quintile cut points were used for men and women. In an analysis using sex-specific cut points, the results presented in the manuscript were unchanged.

+ Mean and (standard deviation).

**Table 2:** Top Five Primary Dietary Sources for Micronutrients in NIH-AARP Diet and Health Study for Men and Women Combined.

Micronutrient+Mean (SD) Intake	Primary Source (%)	Secondary Source (%)	Tertiary Source (%)	Fourth Source (%)	Fifth Source (%)
Selenium: 94±42.9	Bread/Rolls (13.9)	Pasta (6.7)	Tuna (4.8)	Fish - Not Fried (4.1)	Eggs (4.0)
Vitamin C: 157.0±103.7	Orange/GrapefruitJuice (29.1)	Orange/Tangelos (7.9)	Broccoli (7.3)	Other Juice (5.1)	Grapefruit (4.5)
Beta-carotene:4,234.3±3,767.6	Carrots (36.8)	Sweet Potatoes (12.4)	Spinach/Greens (9.8)	Vegetable Medley (5.3)	Broccoli (2.9)
Calcium: 766.5±429.1	Milk –1 & 2% (11.2)	Milk - Skim (10.8)	Milk - In Cereal (9.0)	Bread/Rolls (5.4)	Cereal (3.7)
Folate: 411.4±182.2	Cereal (14.5)	Orange/GrapefruitJuice (10.8)	Lettuce (4.9)	Bread/Rolls (4.7)	Spinach/Greens (4.1)
Magnesium: 326.2±129.7	Coffee (11.3)	Bread/Rolls (5.6)	Cereal (5.4)	Orange/Grapefruit Juice (4.2)	Bananas (3.5)
Vitamin E: 8.8±4.6	Cereal (10.2)	Salad Dressing (7.5)	Oils (3.8)	Nuts/Seed -Whole (3.7)	Tomato Sauces w/Meat (3.6)
Zinc: 10.4±4.8	Cereal (11.0)	Beef - Steak (4.8)	Bread/Rolls (4.4)	Beef - Burger (4.2)	Beef - Meatball (3.3)

+ Micronutrients measured as followed: Selenium (mcg/day), Betacarotene (mcg/day), Calcium (mg/day), Folate (mcg/day), Magnesium (mg/day), Vitamin C (mg/day), Vitamin D (mcg/day), Vitamin E (mg/day), Zinc (mg/day). Vitamin D food sources not available.

Table 3 presents the association between dietary micronutrient intake and thyroid cancer risk. After controlling for potential confounders there were no statistically significant associations between increasing quintile of selenium intake and risk of thyroid cancer (HR_Q5 vs Q1_, 1.23; 95% CI, 0.92–1.65; P_trend_, 0.26). There was a statistically significant increase in risk of thyroid cancer for the highest versus lowest quintile of vitamin C intake in our multivariable model prior to adjusting for antioxidant intake (HR_Q5 vs Q1_, 1.34; 95% CI, 1.02–1.76; P_trend_, <0.01), but not after (HR_Q5 vs Q1_, 1.35; 95% CI, 0.99–1.84; P_trend_, 0.09). Although we observed evidence of a statistically significant positive linear trend for vitamin C, the HRs were highest for the fourth versus first quintile and were slightly attenuated for the highest quintile. For the remaining micronutrients in our study there was no clear evidence of a positive or negative association, or of a linear trend, between intake of any micronutrient and thyroid cancer risk.

**Table 3:** Hazard Ratios (HRs) and corresponding 95% confidence intervals (CIs) for total thyroid cancer by quintile of micronutrient intake in men and women combined in The NIH-AARP Diet and Health Study.

Selenium	Q1	Q2	Q3	Q4	Q5	P _trend_
Median Intake	7.05	7.64	8.03	8.41	8.93	
Number of Cases	138	131	115	97	111	
Age-adjusted HR^1^ (95% CI)	1.00 (ref)	0.96 (0.75, 1.22)	0.85 (0.66, 1.09)	0.72 (0.56, 0.94)	0.83 (0.65, 1.07)	0.03
Multivariable HR^2^ (95% CI)	1.00 (ref)	1.00 (0.79, 1.28)	0.99 (0.76, 1.29)	1.00 (0.75, 1.33)	1.23 (0.92, 1.65)	0.26
Multivariable HR^3^ (95% CI)	1.00 (ref)	1.07 (0.83, 1.36)	1.07 (0.81, 1.40)	1.10 (0.82, 1.48)	1.35 (0.99, 1.84)	0.09
**Vitamin C**	**Q1**	**Q2**	**Q3**	**Q4**	**Q5**	**P _trend_**
Median Intake	7	8.41	9.36	10.28	11.67	
Number of Cases	36	43	46	67	63	
Age-adjusted HR^1^ (95% CI)	1.00 (ref)	1.19 (0.77, 1.86)	1.27 (0.82, 1.96)	1.80 (1.20, 2.70)	1.57 (1.04, 2.36)	0.01
Multivariable HR^2^ (95% CI)	1.00 (ref)	1.03 (0.78, 1.36)	1.07 (0.81, 1.42)	1.43 (1.09, 1.86)	1.34 (1.02, 1.76)	<0.01
Multivariable HR^3^ (95% CI)	1.00 (ref)	1.03 (0.77, 1.37)	1.11 (0.82, 1.49)	1.47 (1.09, 1.98)	1.46 (1.05, 2.04)	<0.01
**Betacarotene**	**Q1**	**Q2**	**Q3**	**Q4**	**Q5**	**P _trend_**
Median Intake	8.67	9.38	9.89	10.43	11.3	
Number of Cases	111	116	107	123	132	
Age-adjusted HR^1^ (95% CI)	1.00 (ref)	1.03 (0.80, 1.34)	0.95 (0.73, 1.24)	1.09 (0.84, 1.40)	1.16 (0.90, 1.50)	0.2
Multivariable HR^2^ (95% CI)	1.00 (ref)	1.00 (0.77, 1.30)	0.87 (0.67, 1.15)	1.01 (0.78, 1.32)	1.07 (0.83, 1.39)	0.6
Multivariable HR^3^ (95% CI)	1.00 (ref)	0.93 (0.71, 1.22)	0.78 (0.59, 1.04)	0.87 (0.66, 1.16)	0.89 (0.65, 1.20)	0.38
**Calcium**	**Q1**	**Q2**	**Q3**	**Q4**	**Q5**	**P _trend_**
Median Intake	8.67	9.38	9.89	10.43	11.3	
Number of Cases	111	116	107	123	132	
Age-adjusted HR^1^ (95% CI)	1.00 (ref)	1.03 (0.80, 1.34)	0.95(0.73, 1.24)	1.09 (0.84, 1.40)	1.16(0.90, 1.50)	0.2
Multivariable HR^2^ (95% CI)	1.00 (ref)	0.94 (0.71, 1.23)	0.92 (0.69, 1.23)	1.15 (0.85, 1.56)	1.08 (0.74, 1.58)	0.63
Multivariable HR^3^ (95% CI)	1.00 (ref)	0.92 (0.69, 1.21)	0.90 (0.66, 1.21)	1.11 (0.81, 1.53)	1.00 (0.67, 1.48)	0.94
**Folate**	**Q1**	**Q2**	**Q3**	**Q4**	**Q5**	**P _trend_**
Median Intake	11.7	12.58	13.17	13.78	14.72	
Number of Cases	121	115	129	110	117	
Age-adjusted HR^1^ (95% CI)	1.00 (ref)	0.94 (0.73, 1.22)	1.06 (0.83, 1.36)	0.90 (0.70, 1.17)	0.96 (0.75, 1.24)	0.7
Multivariable HR^2^ (95% CI)	1.00 (ref)	0.94 (0.73, 1.22)	1.17 (0.91, 1.51)	1.04 (0.79, 1.36)	1.27 (0.97, 1.67)	0.06
Multivariable HR^3^ (95% CI)	1.00 (ref)	0.94 (0.73, 1.22)	1.17 (0.91, 1.51)	1.04 (0.79, 1.36)	1.27 (0.97, 1.67)	0.06
**Vitamin E**	**Q1**	**Q2**	**Q3**	**Q4**	**Q5**	**P _trend_**
Median Intake	1.85	2.09	2.26	2.43	2.71	
Number of Cases	125	133	126	108	97	
Age-adjusted HR^1^ (95% CI)	1.00 (ref)	1.06 (0.83, 1.35)	1.01 (0.79, 1.29)	0.87 (0.67, 1.12)	0.78 (0.60, 1.02)	0.03
Multivariable HR^2^ (95% CI)	1.00 (ref)	1.09 (0.84, 1.39)	1.12 (0.87, 1.45)	1.05 (0.80, 1.37)	0.99 (0.75, 1.31)	0.92
Multivariable HR^3^ (95% CI)	1.00 (ref)	1.07 (0.83, 1.38)	1.11 (0.85, 1.44)	1.02 (0.77, 1.35)	0.93 (0.69, 1.25)	0.58
**Vitamin D**	**Q1**	**Q2**	**Q3**	**Q4**	**Q5**	**P _trend_**
Median Intake	0.58	1.14	1.51	1.89	2.46	
Number of Cases	114	116	135	117	109	
Age-adjusted HR^1^ (95% CI)	1.00 (ref)	1.04 (0.80, 1.35)	1.19 (0.91, 1.57)	0.99 (0.73, 1.34)	0.90 (0.62, 1.31)	0.77
Multivariable HR^2^ (95% CI)	1.00 (ref)	1.10 (0.84, 1.44)	1.24 (0.94, 1.65)	1.09 (0.79, 1.49)	1.03 (0.70, 1.50)	0.72
Multivariable HR^3^ (95% CI)	1.00 (ref)	1.08 (0.82, 1.43)	1.25 (0.93, 1.66)	1.08 (0.78, 1.49)	1.03 (0.70, 1.53)	0.71
**Magnesium**	**Q1**	**Q2**	**Q3**	**Q4**	**Q5**	**P _trend_**
Median Intake	10.1	10.72	11.11	11.49	12.03	
Number of Cases	145	115	116	113	100	
Age-adjusted HR^1^ (95% CI)	1.00 (ref)	0.79 (0.62, 1.01)	0.80 (0.63, 1.02)	0.79 (0.61, 1.00)	0.70 (0.54, 0.90)	<0.01
Multivariable HR^2^ (95% CI)	1.00 (ref)	0.85 (0.66, 1.09)	0.96 (0.74, 1.23)	1.03 (0.79, 1.34)	1.00 (0.76, 1.33)	0.66
Multivariable HR^3^ (95% CI)	1.00 (ref)	0.79 (0.61, 1.03)	0.86 (0.66, 1.14)	0.88 (0.66, 1.19)	0.80 (0.57, 1.13)	0.33
**Zinc**	**Q1**	**Q2**	**Q3**	**Q4**	**Q5**	**P _trend_**
Median Intake	2.24	2.54	2.75	2.95	3.24	
Number of Cases	146	138	106	104	98	
Age-adjusted HR^1^ (95% CI)	1.00 (ref)	0.95 (0.75, 1.20)	0.74 (0.57, 0.94)	0.73 (0.57, 0.94)	0.69 (0.54, 0.90)	<0.01
Multivariable HR^2^ (95% CI)	1.00 (ref)	0.96 (0.76, 1.22)	0.85 (0.66, 1.11)	0.95 (0.72, 1.25)	0.98 (0.73, 1.32)	0.96
Multivariable HR^3^ (95% CI)	1.00 (ref)	0.94 (0.74, 1.21)	0.82 (0.62, 1.08)	0.87 (0.64, 1.18)	0.89 (0.63, 1.25)	0.36

1 Adjusted for entry age.

2 Adjusted for entry age, sex (overall), calories, smoking status, race, education, BMI, and physical activity.

3 Additionally adjusted for vitamin C, vitamin E, beta-carotene, and folate.

We also evaluated the associations between micronutrient intake and thyroid cancer separately by sex because of the higher incidence of thyroid cancer in women and suspected differences in the etiology of thyroid cancer by sex [40] (Tables S1 and S2). Although for some micronutrients, such as vitamin C, the strength and direction of the association appeared to differ by sex there were no statistically significant interactions by sex. Results restricted to papillary or follicular thyroid cancer, the two most common subtypes of thyroid cancer, were largely similar to the overall analysis (Tables S3–S8).

When we restricted the analysis to only participants with complete covariate information the results did not vary substantially from the presented data with an indicator variable for unknown values. There was also no indication that additionally adjusting for intake of the antioxidants vitamin C, vitamin E, beta-carotene, and folate in our multivariable model had an impact on the association with selenium or any other micronutrient (multivariable model 2 vs. 3). Finally there was no evidence that using sex-specific quintiles of micronutrient intake in men and women had a substantial impact on the results presented.

## Discussion

Using a large prospective cohort study, we observed no evidence of an association between quintile of selenium intake and incidence of total thyroid cancer or the papillary and follicular subtypes. This was the first prospective study on this topic. We observed a suggestion of a positive linear relationship between increasing quintile of vitamin C and the risk of total thyroid cancer and the papillary and follicular subtypes, but no evidence of an association between thyroid cancer risk and calcium, folate, vitamin E, vitamin D, magnesium, or zinc intakes.

Selenium is a biologically important micronutrient shown to have a preventive effect for cancers other than thyroid [27]. Selenium assists in the production and proper function of thyroid hormones and has antioxidant properties [23]–[26]. Several limitations of our study may have contributed to the null results observed for selenium. Using an FFQ to determine dietary selenium intake may have resulted in exposure misclassification because the selenium content of soil varies largely by geographic region [41], [42], resulting in a difference in the accumulation of selenium in animals and plant products and in the selenium content of foods. An additional limitation of our study is that measurement error in dietary micronutrient intake, which was likely non-differential, may have attenuated the associations of interest. Use of bio-specimens in future studies would allow for more accurate measurement of an individual’s selenium intake. Measuring plasma selenium is a good indicator of recent intake [43], [44] although plasma selenium concentrations are not useful for determining long term intake and recent infections can influence plasma selenium levels [45]. Toenail clippings have been used to indicate long term selenium intake and are useful in investigations between selenium and chronic diseases [45], [46].

Another limiting factor of using an FFQ in our study population was that the participants were not asked about every possible contributor to dietary selenium. For example, dietary consumption of Brazilian nuts, the food with the highest selenium content [47], was not assessed. Intake of fresh halibut and sardines, two other food sources with high selenium content were also not assessed. A study of the major food sources of antioxidants in U.S. adults [48] showed that Brazilian nuts, halibut, and sardines are not major dietary contributors of selenium in the U.S. diet and that the dietary contributors of selenium included in our study are representative of the major sources of selenium in the U.S. diet. Therefore, the impact of not having information on Brazilian nut consumption in our study was likely minimal. Further, the bio-availability of selenium from different food sources varies, and wheat, which was the highest contributor of dietary selenium in our study, has a high bio-availability [49].

In our analysis of other micronutrients and their association with thyroid cancer we saw evidence for an association with intake of vitamin C only. Higher intake of vitamin C appeared to be positively associated with thyroid cancer risk. Biologically, vitamin C has been shown to improve and mediate abnormalities seen in the levels of thyroid hormones and thyroid stimulating hormone in the serum of humans [50] and rats [51]. Previous studies have indicated both a positive and negative association between increased vitamin C intake and thyroid cancer [28]. A recent case-control study by Jung et al. indicated that vitamin C intake and citrus consumption in controls was higher than in individuals with either benign or malignant thyroid cancer, although the difference was not statistically significant [13]. A positive association between citrus consumption and thyroid cancer was also seen by Xiao et al. and appeared to be driven by the intake of orange and grapefruit juice [52]. Orange and grapefruit juices were the primary contributors of vitamin C intake in our study. Xiao et al. suggest some individuals may have included artificially flavored drinks in their report of orange and grapefruit juice consumption, leading to misclassification and potentially a false positive association.

It is also possible that residual confounding may have contributed to our observed association between increasing quintile of vitamin C intake and thyroid cancer risk. Participants in the highest quintile of vitamin C intake had higher education, which has been associated with healthcare utilization and increased rates of thyroid cancer diagnosis [53], [54]. Additionally, individuals in the highest quintile of vitamin C intake had greater physical activity and lower caloric intake, which are characteristics associated with a health conscious lifestyle. A healthier lifestyle, again, may correspond to greater healthcare utilization and increased opportunity for thyroid cancer detection. Although we did control for these factors in our analysis it is possible that they were measured imperfectly or that we were unable to fully capture aspects of a healthy lifestyle or health consciousness that could influence detection. It seems unlikely, however, that increased detection would occur only in men and impact only the results for vitamin C.

Our study has several strengths. While previous studies have utilized case-control designs this study is the first to examine the association between selenium, and other micronutrient, intake and thyroid cancer using a large prospective cohort design, greatly reducing the possibility of recall and selection biases [55]. Furthermore, there was generally complete follow-up of study participants to ascertain outcomes and a relatively large number of thyroid cancer cases, which allowed us to analyze thyroid cancer by histologic subtype. Additionally we had covariate information that allowed us to adjust for potential confounders in our multivariable analysis of this study population. Using quintiles of selenium intake allowed for a natural comparison group, as the lowest unadjusted quintile of intake for selenium (less than 47 mcg/day) was the only group which fell below the recommended daily intake for men and women of 55 mcg/day [56].

It is possible that exposure to essential micronutrients earlier in life, or over the course of an individual’s lifetime, may be more important in determining thyroid cancer risk. Although the NIH-AARP study utilized follow-up questionnaires that asked about diet at different points in the participant’s life, the follow-up questions were not designed to assess selenium and therefore were not used in this analysis. Future studies with information on dietary intake over the span of a person’s life could add valuable information to dietary studies on thyroid cancer risk. Finally, because the FFQ in this study was not designed to measure iodine consumption, we did not have information on daily iodine intake, which is important for thyroid function and may be a potential important confounding variable. However, iodine deficiency and the subsequent impact of not controlling for iodine intake on our study results is likely minimal, as iodine fortification of salt and other foods has occurred in the United States since the 1920’s [57].

In conclusion we observed no evidence of an association between thyroid cancer and quintile of selenium intake. Given the unexpected positive association observed for vitamin C, further evaluation of the association between vitamin C and thyroid cancer in other prospective cohorts is warranted. Valuable follow-up studies of selenium and thyroid cancer risk could evaluate selenium intake and intake of other micronutrients more objectively, such as by use of biomarkers.

## Supporting Information

Table S1 **Hazard Ratios (HRs) and corresponding 95% confidence intervals (CIs) for total thyroid cancer by quintile of micronutrient intake among men in The NIH-AARP Diet and Health Study.**
(DOCX)

Table S2 **Hazard Ratios (HRs) and corresponding 95% confidence intervals (CIs) for total thyroid cancer by quintile of micronutrient intake among women in The NIH-AARP Diet and Health Study.**
(DOCX)

Table S3 **Hazard Ratios (HRs) and corresponding 95% confidence intervals (CIs) for papillary thyroid cancer by quintile of micronutrient intake among men and women combined in The NIH-AARP Diet and Health Study.**
(DOCX)

Table S4 **Hazard Ratios (HRs) and corresponding 95% confidence intervals (CIs) for papillary thyroid cancer by quintile of micronutrient intake among men in The NIH-AARP Diet and Health Study.**
(DOCX)

Table S5 **Hazard Ratios (HRs) and corresponding 95% confidence intervals (CIs) for papillary thyroid cancer by quintile of micronutrient intake among women in The NIH-AARP Diet and Health Study.**
(DOCX)

Table S6 **Hazard Ratios (HRs) and corresponding 95% confidence intervals (CIs) for total thyroid cancer by quintile of micronutrient intake among men in The NIH-AARP Diet and Health Study.**
(DOCX)

Table S7 **Hazard Ratios (HRs) and corresponding 95% confidence intervals (CIs) for follicular thyroid cancer by quintile of micronutrient intake among men in The NIH-AARP Diet and Health Study.**
(DOCX)

Table S8 **Hazard Ratios (HRs) and corresponding 95% confidence intervals (CIs) for follicular thyroid cancer by quintile of micronutrient intake among women in The NIH-AARP Diet and Health Study.**
(DOCX)
