# Supplementary material for: The Association between Selenium and Other Micronutrients and Thyroid Cancer Incidence in the NIH-AARP Diet and Health Study
Source: PLoS One. 2014 Oct 20;9(10):e110886. doi: 10.1371/journal.pone.0110886 (PMC4203851; doi:10.1371/journal.pone.0110886)
Supplement: Table S1 — Hazard Ratios (HRs) and corresponding 95% confidence intervals (CIs) for total thyroid cancer by quintile of micronutrient intake among men in The NIH-AARP Diet and Health Study. (DOCX) [file pone.0110886.s001.docx]

**Table S1 – Hazard Ratios (HRs) and corresponding 95% confidence intervals (CIs) for total thyroid cancer by quintile of micronutrient intake among men in The NIH-AARP Diet and Health Study:**

| **Selenium** | **Q1** | **Q2** | **Q3** | **Q4** | **Q5** | **P _trend_** |
| --- | --- | --- | --- | --- | --- | --- |
| Median Intake | 7.05 | 7.64 | 8.03 | 8.41 | 8.93 |  |
| Number of Cases | 19 | 25 | 49 | 68 | 96 |  |
| Age-adjusted HR^1^ (95% CI) | 1.00 (ref) | 0.97 (0.72, 1.29) | 0.88 (0.65, 1.18) | 0.76 (0.55, 1.03) | 0.73 (0.54, 1.00) | 0.02 |
| Multivariable HR^2^ (95% CI) | 1.00 (ref) | 0.69 (0.38, 1.25) | 0.82 (0.48, 1.40) | 0.91 (0.55, 1.53) | 1.08 (0.66, 1.77) | 0.13 |
| Multivariable HR^3^ (95% CI) | 1.00 (ref) | 0.74 (0.39, 1.38) | 0.90 (0.51, 1.59) | 1.04 (0.60, 1.79) | 1.23 (0.71, 2.12) | 0.05 |
| **Vitamin C** | **Q1** | **Q2** | **Q3** | **Q4** | **Q5** | **P _trend_** |
| Median Intake | 7 | 8.41 | 9.36 | 10.28 | 11.67 |  |
| Number of Cases | 36 | 43 | 46 | 67 | 63 |  |
| Age-adjusted HR^1^ (95% CI) | 1.00 (ref) | 1.19 (0.77, 1.86) | 1.27 (0.82, 1.96) | 1.80 (1.20, 2.70) | 1.57 (1.20, 2.70) | 0.01 |
| Multivariable HR^2^ (95% CI) | 1.00 (ref) | 1.11 (0.71, 1.73) | 1.11 (0.71, 1.74) | 1.67 (1.10, 2.52) | 1.50 (0.98, 2.28) | 0.01 |
| Multivariable HR^3^ (95% CI) | 1.00 (ref) | 1.09 (0.69, 1.72) | 1.10 (0.69, 1.77) | 1.61 (1.01, 2.57) | 1.55 (0.93, 2.57) | 0.03 |
| **Betacarotene** | **Q1** | **Q2** | **Q3** | **Q4** | **Q5** | **P _trend_** |
| Median Intake | 8.67 | 9.38 | 9.89 | 10.43 | 11.3 |  |
| Number of Cases | 47 | 49 | 50 | 57 | 52 |  |
| Age-adjusted HR^1^ (95% CI) | 1.00 (ref) | 1.04 (0.70, 1.55) | 1.09 (0.73, 1.62) | 1.29 (0.87, 1.89) | 1.27 (0.86, 1.89) | 0.13 |
| Multivariable HR^2^ (95% CI) | 1.00 (ref) | 0.97 (0.65, 1.46) | 1.01 (0.67, 1.51) | 1.18 (0.79, 1.75) | 1.20 (0.80, 1.01) | 0.25 |
| Multivariable HR^3^ (95% CI) | 1.00 (ref) | 0.90 (0.60, 1.37) | 0.88 (0.58, 1.35) | 0.96 (0.63, 1.47) | 0.93 (0.58, 1.49) | 0.88 |
| **Calcium** | **Q1** | **Q2** | **Q3** | **Q4** | **Q5** | **P _trend_** |
| Median Intake | 8.67 | 9.38 | 9.89 | 10.43 | 11.3 |  |
| Number of Cases | 47 | 49 | 50 | 57 | 52 |  |
| Age-adjusted HR^1^ (95% CI) | 1.00 (ref) | 1.04 (0.70, 1.55) | 1.09 (0.73, 1.62) | 1.29 (0.87, 1.89) | 1.27 (0.86, 1.89) | 0.13 |
| Multivariable HR^2^ (95% CI) | 1.00 (ref) | 1.24 (0.77, 2.02) | 1.25 (0.76, 2.06) | 1.55 (0.93, 2.59) | 1.39 (0.76, 2.54) | 0.33 |
| Multivariable HR^3^ (95% CI) | 1.00 (ref) | 1.16 (0.70, 1.90) | 1.14 (0.68, 1.93) | 1.39 (0.81, 2.38) | 1.15 (0.61, 2.17) | 0.76 |
| **Folate** | **Q1** | **Q2** | **Q3** | **Q4** | **Q5** | **P _trend_** |
| Median Intake | 11.72 | 12.58 | 13.17 | 13.78 | 14.72 |  |
| Number of Cases | 25 | 36 | 51 | 58 | 87 |  |
| Age-adjusted HR^1^ (95% CI) | 1.00 (ref) | 1.07 (0.64, 1.78) | 1.25 (0.77, 2.01) | 1.19 (0.74, 1.90) | 1.55 (0.99, 2.42) | 0.02 |
| Multivariable HR^2^ (95% CI) | 1.00 (ref) | 1.04 (0.63, 1.74) | 1.19 (0.73, 1.93) | 1.11 (0.69, 1.80) | 1.52 (0.96, 2.40) | 0.04 |
| Multivariable HR^3^ (95% CI) | 1.00 (ref) | 0.90 (0.53, 1.53) | 0.92 (0.55, 1.56) | 0.83 (0.48, 1.42) | 1.02 (0.58, 1.81) | 0.86 |
| **Vitamin E** | **Q1** | **Q2** | **Q3** | **Q4** | **Q5** | **P _trend_** |
| Median Intake | 1.85 | 2.09 | 2.26 | 2.43 | 2.71 |  |
| Number of Cases | 29 | 46 | 48 | 67 | 66 |  |
| Age-adjusted HR^1^ (95% CI) | 1.00 (ref) | 1.25 (0.79, 1.99) | 1.04 (0.65, 1.65) | 1.24 (0.80, 1.92) | 1.12 (0.72, 1.74) | 0.75 |
| Multivariable HR^2^ (95% CI) | 1.00 (ref) | 1.18 (0.74, 1.90) | 0.98 (0.61, 1.57) | 1.17 (0.75, 1.82) | 1.11 (0.71, 1.73) | 0.73 |
| Multivariable HR^3^ (95% CI) | 1.00 (ref) | 1.11 (0.68, 1.80) | 0.89 (0.55, 1.45) | 1.03 (0.64, 1.66) | 0.95 (0.59, 1.54) | 0.73 |
| **Vitamin D** | **Q1** | **Q2** | **Q3** | **Q4** | **Q5** | **P _trend_** |
| Median Intake | 0.58 | 1.14 | 1.51 | 1.89 | 2.46 |  |
| Number of Cases | 31 | 46 | 56 | 64 | 60 |  |
| Age-adjusted HR^1^ (95% CI) | 1.00 (ref) | 1.11 (0.70, 1.76) | 1.11 (0.69, 1.77) | 1.07 (0.66, 1.75) | 0.95 (0.54, 1.68) | 0.75 |
| Multivariable HR^2^ (95% CI) | 1.00 (ref) | 1.13 (0.71, 1.82) | 1.11 (0.69, 1.80) | 1.07 (0.65, 1.77) | 0.94 (0.52, 1.67) | 0.71 |
| Multivariable HR^3^ (95% CI) | 1.00 (ref) | 1.16 (0.71, 1.88) | 1.16 (0.71, 1.91) | 1.11 (0.66, 1.87) | 1.02 (0.56, 1.86) | 0.92 |
| **Magnesium** | **Q1** | **Q2** | **Q3** | **Q4** | **Q5** | **P _trend_** |
| Median Intake | 10.14 | 10.72 | 11.11 | 11.49 | 12.03 |  |
| Number of Cases | 21 | 38 | 51 | 67 | 79 |  |
| Age-adjusted HR^1^ (95% CI) | 1.00 (ref) | 1.19 (0.70, 2.02) | 1.23 (0.74, 2.05) | 1.33 (0.82, 2.18) | 1.34 (0.83, 2.17) | 0.21 |
| Multivariable HR^2^ (95% CI) | 1.00 (ref) | 1.17 (0.68, 2.02) | 1.23 (0.73, 2.06) | 1.29 (0.78, 2.14) | 1.30 (0.79, 2.13) | 0.30 |
| Multivariable HR^3^ (95% CI) | 1.00 (ref) | 1.13 (0.64, 1.98) | 1.11 (0.64, 1.93) | 1.09 (0.62, 1.91) | 1.00 (0.56, 1.80) | 0.81 |
| **Zinc** | **Q1** | **Q2** | **Q3** | **Q4** | **Q5** | **P _trend_** |
| Median Intake | 2.24 | 2.54 | 2.75 | 2.95 | 3.24 |  |
| Number of Cases | 17 | 31 | 45 | 77 | 87 |  |
| Age-adjusted HR^1^ (95% CI) | 1.00 (ref) | 1.08 (0.60, 1.95) | 1.02 (0.58, 1.78) | 1.32 (0.78, 2.23) | 1.34 (0.80, 2.25) | 0.10 |
| Multivariable HR^2^ (95% CI) | 1.00 (ref) | 0.95 (0.52, 1.72) | 0.90 (0.52, 1.58) | 1.10 (0.65, 1.88) | 1.15 (0.68, 1.95) | 0.26 |
| Multivariable HR^3^ (95% CI) | 1.00 (ref) | 0.95 (0.50, 1.79) | 0.85 (0.46, 1.58) | 0.99 (0.54, 1.83) | 0.98 (0.52, 1.84) | 0.80 |

^1^ Adjusted for entry age ^2^Adjusted for entry age, sex (overall), calories, smoking status, race, education, BMI, and physical activity ^3^Additionally adjusted for

vitamin C, vitamin E, beta-carotene, and folate
