# Supplementary material for: The Association between Selenium and Other Micronutrients and Thyroid Cancer Incidence in the NIH-AARP Diet and Health Study
Source: PLoS One. 2014 Oct 20;9(10):e110886. doi: 10.1371/journal.pone.0110886 (PMC4203851; doi:10.1371/journal.pone.0110886)
Supplement: Table S2 — Hazard Ratios (HRs) and corresponding 95% confidence intervals (CIs) for total thyroid cancer by quintile of micronutrient intake among women in The NIH-AARP Diet and Health Study. (DOCX) [file pone.0110886.s002.docx]

**Table S2 – Hazard Ratios (HRs) and corresponding 95% confidence intervals (CIs) for total thyroid cancer by quintile of micronutrient intake among women in The NIH-AARP Diet and Health Study:**

| **Selenium** | **Q1** | **Q2** | **Q3** | **Q4** | **Q5** | **P _trend_** |
| --- | --- | --- | --- | --- | --- | --- |
| Median Intake | 7.05 | 7.64 | 8.03 | 8.41 | 8.93 |  |
| Number of Cases | 119 | 106 | 66 | 29 | 15 |  |
| Age-adjusted HR^1^ (95% CI) | 1.00 (ref) | 0.89 (0.50, 1.58) | 0.90 (0.51, 1.59) | 0.79 (0.44, 1.43) | 1.05 (0.60, 1.83) | 0.67 |
| Multivariable HR^2^ (95% CI) | 1.00 (ref) | 1.11 (0.85, 1.45) | 1.06 (0.78, 1.44) | 0.90 (0.60, 1.37) | 1.12 (0.65, 1.93) | 0.90 |
| Multivariable HR^3^ (95% CI) | 1.00 (ref) | 1.17 (0.89, 1.54) | 1.14 (0.82, 1.56) | 0.98 (0.64, 1.50) | 1.14 (0.65, 2.02) | 0.62 |
| **Vitamin C** | **Q1** | **Q2** | **Q3** | **Q4** | **Q5** | **P _trend_** |
| Median Intake | 7 | 8.41 | 9.36 | 10.28 | 11.67 |  |
| Number of Cases | 58 | 62 | 68 | 80 | 66 |  |
| Age-adjusted HR^1^ (95% CI) | 1.00 (ref) | 1.04 (0.73, 1.49) | 1.14 (0.80, 1.62) | 1.39 (0.99, 1.95) | 1.29 (0.91, 1.84) | 0.05 |
| Multivariable HR^2^ (95% CI) | 1.00 (ref) | 0.98 (0.68, 1.41) | 1.05 (0.73, 1.50) | 1.28 (0.90, 1.81) | 1.25 (0.87, 1.80) | 0.09 |
| Multivariable HR^3^ (95% CI) | 1.00 (ref) | 0.99 (0.68, 1.44) | 1.11 (0.76, 1.62) | 1.37 (0.93, 2.03) | 1.40 (0.90, 2.19) | 0.06 |
| **Betacarotene** | **Q1** | **Q2** | **Q3** | **Q4** | **Q5** | **P _trend_** |
| Median Intake | 8.67 | 9.38 | 9.89 | 10.43 | 11.3 |  |
| Number of Cases | 64 | 67 | 57 | 66 | 80 |  |
| Age-adjusted HR^1^ (95% CI) | 1.00 (ref) | 1.02 (0.72, 1.43) | 0.82 (0.57, 1.17) | 0.89 (0.63, 1.26) | 0.97 (0.70, 1.34) | 0.74 |
| Multivariable HR^2^ (95% CI) | 1.00 (ref) | 1.02 (0.72, 1.44) | 0.78 (0.54, 1.12) | 0.90 (0.63, 1.28) | 1.00 (0.71, 1.41) | 0.82 |
| Multivariable HR^3^ (95% CI) | 1.00 (ref) | 0.95 (0.67, 1.36) | 0.70 (0.48, 1.03) | 0.81 (0.56, 1.19) | 0.87 (0.59, 1.30) | 0.36 |
| **Calcium** | **Q1** | **Q2** | **Q3** | **Q4** | **Q5** | **P _trend_** |
| Median Intake | 8.67 | 9.38 | 9.89 | 10.43 | 11.3 |  |
| Number of Cases | 64 | 67 | 57 | 66 | 80 |  |
| Age-adjusted HR^1^ (95% CI) | 1.00 (ref) | 1.02 (0.72, 1.43) | 0.82 (0.57, 1.17) | 0.89 (0.63, 1.26) | 0.97 (0.70, 1.34) | 0.74 |
| Multivariable HR^2^ (95% CI) | 1.00 (ref) | 0.82 (0.59, 1.15) | 0.79 (0.55, 1.15) | 1.00 (0.67, 1.48) | 1.00 (0.60, 1.65) | 0.87 |
| Multivariable HR^3^ (95% CI) | 1.00 (ref) | 0.83 (0.59, 1.17) | 0.79 (0.54, 1.17) | 1.00 (0.66, 1.52) | 0.98 (0.57, 1.67) | 0.83 |
| **Folate** | **Q1** | **Q2** | **Q3** | **Q4** | **Q5** | **P _trend_** |
| Median Intake | 11.72 | 12.58 | 13.17 | 13.78 | 14.72 |  |
| Number of Cases | 96 | 79 | 78 | 52 | 30 |  |
| Age-adjusted HR^1^ (95% CI) | 1.00 (ref) | 1.01 (0.75, 1.36) | 1.25 (0.93, 1.69) | 1.13 (0.81, 1.59) | 0.99 (0.65, 1.48) | 0.55 |
| Multivariable HR^2^ (95% CI) | 1.00 (ref) | 0.92 (0.68, 1.25) | 1.21 (0.90, 1.64) | 1.05 (0.74, 1.49) | 1.02 (0.67, 1.55) | 0.49 |
| Multivariable HR^3^ (95% CI) | 1.00 (ref) | 0.86 (0.62, 1.19) | 1.11 (0.79, 1.58) | 0.94 (0.62, 1.43) | 0.89 (0.52, 1.47) | 0.98 |
| **Vitamin E** | **Q1** | **Q2** | **Q3** | **Q4** | **Q5** | **P _trend_** |
| Median Intake | 1.85 | 2.09 | 2.26 | 2.43 | 2.71 |  |
| Number of Cases | 29 | 46 | 48 | 67 | 66 |  |
| Age-adjusted HR^1^ (95% CI) | 1.00 (ref) | 1.25 (0.79, 1.99) | 1.04 (0.65, 1.65) | 1.24 (0.80, 1.92) | 1.12 (0.72, 1.74) | 0.75 |
| Multivariable HR^2^ (95% CI) | 1.00 (ref) | 1.18 (0.74, 1.90) | 0.98 (0.61, 1.57) | 1.17 (0.75, 1.82) | 1.11 (0.71, 1.73) | 0.73 |
| Multivariable HR^3^ (95% CI) | 1.00 (ref) | 1.11 (0.68, 1.80) | 0.89 (0.55, 1.45) | 1.03 (0.64, 1.66) | 0.95 (0.59, 1.54) | 0.73 |
| **Vitamin D** | **Q1** | **Q2** | **Q3** | **Q4** | **Q5** | **P _trend_** |
| Median Intake | 0.58 | 1.14 | 1.51 | 1.89 | 2.46 |  |
| Number of Cases | 83 | 70 | 79 | 53 | 49 |  |
| Age-adjusted HR^1^ (95% CI) | 1.00 (ref) | 1.09 (0.79, 1.51) | 1.45 (1.03, 2.04) | 1.09 (0.72, 1.65) | 1.13 (0.68, 1.89) | 0.34 |
| Multivariable HR^2^ (95% CI) | 1.00 (ref) | 1.09 (0.78, 1.52) | 1.36 (0.95, 1.94) | 1.07 (0.70, 1.63) | 1.10 (0.65, 1.85) | 0.45 |
| Multivariable HR^3^ (95% CI) | 1.00 (ref) | 1.06 (0.76, 1.49) | 1.34 (0.93, 1.93) | 1.03 (0.67, 1.60) | 1.05 (0.61, 1.81) | 0.57 |
| **Magnesium** | **Q1** | **Q2** | **Q3** | **Q4** | **Q5** | **P _trend_** |
| Median Intake | 10.14 | 10.72 | 11.11 | 11.49 | 12.03 |  |
| Number of Cases | 124 | 77 | 65 | 46 | 21 |  |
| Age-adjusted HR^1^ (95% CI) | 1.00 (ref) | 0.80 (0.60, 1.06) | 0.91 (0.67, 1.22) | 0.95 (0.67, 1.33) | 0.81 (0.51, 1.28) | 0.41 |
| Multivariable HR^2^ (95% CI) | 1.00 (ref) | 0.79 (0.59, 1.05) | 0.92 (0.68, 1.25) | 1.01 (0.72, 1.43) | 0.89 (0.55, 1.42) | 0.76 |
| Multivariable HR^3^ (95% CI) | 1.00 (ref) | 0.74 (0.55, 1.01) | 0.85 (0.60, 1.18) | 0.89 (0.60, 1.32) | 0.71 (0.42, 1.25) | 0.26 |
| **Zinc** | **Q1** | **Q2** | **Q3** | **Q4** | **Q5** | **P _trend_** |
| Median Intake | 2.24 | 2.54 | 2.75 | 2.95 | 3.24 |  |
| Number of Cases | 129 | 107 | 61 | 27 | 11 |  |
| Age-adjusted HR^1^ (95% CI) | 1.00 (ref) | 1.04 (0.81, 1.35) | 0.93 (0.69, 1.26) | 0.85 (0.56, 1.29) | 0.72 (0.39, 1.34) | 0.25 |
| Multivariable HR^2^ (95% CI) | 1.00 (ref) | 0.98 (0.75, 1.28) | 0.87 (0.64, 1.19) | 0.82 (0.54, 1.25) | 0.70 (0.38, 1.30) | 0.75 |
| Multivariable HR^3^ (95% CI) | 1.00 (ref) | 0.98 (0.74, 1.28) | 0.86 (0.61, 1.19) | 0.74 (0.47, 1.17) | 0.70 (0.36, 1.36) | 0.11 |

^1^ Adjusted for entry age ^2^Adjusted for entry age, sex (overall), calories, smoking status, race, education, BMI, and physical activity ^3^Additionally adjusted for

vitamin C, vitamin E, beta-carotene, and folate
