# Supplementary material for: The Association between Selenium and Other Micronutrients and Thyroid Cancer Incidence in the NIH-AARP Diet and Health Study
Source: PLoS One. 2014 Oct 20;9(10):e110886. doi: 10.1371/journal.pone.0110886 (PMC4203851; doi:10.1371/journal.pone.0110886)
Supplement: Table S3 — Hazard Ratios (HRs) and corresponding 95% confidence intervals (CIs) for papillary thyroid cancer by quintile of micronutrient intake among men and women combined in The NIH-AARP Diet and Health Study. (DOCX) [file pone.0110886.s003.docx]

**Table S3 – Hazard Ratios (HRs) and corresponding 95% confidence intervals (CIs) for papillary thyroid cancer by quintile of micronutrient intake among men and women combined in The NIH-AARP Diet and Health Study:**

| **Selenium** | **Q1** | **Q2** | **Q3** | **Q4** | **Q5** | **P _trend_** |
| --- | --- | --- | --- | --- | --- | --- |
| Median Intake | 7.05 | 7.64 | 8.03 | 8.41 | 8.93 |  |
| Number of Cases | 95 | 91 | 82 | 70 | 68 |  |
| Age-adjusted HR^1^ (95% CI) | 1.00 (ref) | 0.97 (0.72, 1.29) | 0.88 (0.65, 1.18) | 0.76, 0.55, 1.03) | 0.73 (0.54, 1.00) | 0.02 |
| Multivariable HR^2^ (95% CI) | 1.00 (ref) | 1.02 (0.76, 1.37) | 1.04 (0.76, 1.43) | 1.11 (0.79, 1.56) | 1.20 (0.83, 1.72) | 0.32 |
| Multivariable HR^3^ (95% CI) | 1.00 (ref) | 1.11 (0.82, 1.50) | 1.14 (0.83, 1.58) | 1.26 (0.89, 1.79) | 1.35 (0.92, 1.98) | 0.11 |
| **Vitamin C** | **Q1** | **Q2** | **Q3** | **Q4** | **Q5** | **P _trend_** |
| Median Intake | 7 | 8.41 | 9.36 | 10.27 | 11.67 |  |
| Number of Cases | 20 | 24 | 29 | 52 | 37 |  |
| Age-adjusted HR^1^ (95% CI) | 1.00 (ref) | 1.21 (0.67, 2.19) | 1.46 (0.83, 2.59) | 2.58 (1.54, 4.32) | 1.7 (0.98, 2.93) | <0.01 |
| Multivariable HR^2^ (95% CI) | 1.00 (ref) | 1.10 (0.77, 1.58) | 1.35 (0.96, 1.92) | 1.93 (1.39, 2.69) | 1.47 (1.03, 2.09) | <0.01 |
| Multivariable HR^3^ (95% CI) | 1.00 (ref) | 1.10 (0.76, 1.59) | 1.41 (0.98, 2.04) | 2.02 (1.40, 2.91) | 1.60 (1.05, 2.43) | <0.01 |
| **Betacarotene** | **Q1** | **Q2** | **Q3** | **Q4** | **Q5** | **P _trend_** |
| Median Intake | 8.67 | 9.38 | 9.89 | 10.43 | 11.3 |  |
| Number of Cases | 77 | 75 | 77 | 84 | 90 |  |
| Age-adjusted HR^1^ (95% CI) | 1.00 (ref) | 0.97 (0.71, 1.34) | 1.00 (0.73, 1.37) | 1.09 (0.80, 1.47) | 1.16 (0.86, 1.58) | 0.23 |
| Multivariable HR^2^ (95% CI) | 1.00 (ref) | 0.94 (0.68, 1.30) | 0.89 (0.65, 1.24) | 1.01 (0.75, 1.39) | 1.04 (0.76, 1.43) | 0.70 |
| Multivariable HR^3^ (95% CI) | 1.00 (ref) | 0.86 (0.62, 1.20) | 0.79 (0.56, 1.10) | 0.84 (0.60, 1.18) | 0.82 (0.57, 1.18) | 0.31 |
| **Calcium** | **Q1** | **Q2** | **Q3** | **Q4** | **Q5** | **P _trend_** |
| Median Intake | 8.67 | 9.38 | 9.89 | 10.43 | 11.3 |  |
| Number of Cases | 77 | 75 | 77 | 84 | 90 |  |
| Age-adjusted HR^1^ (95% CI) | 1.00 (ref) | 0.97 (0.71, 1.34) | 1.00 (0.73, 1.37) | 1.09 (0.80, 1.48) | 1.16 (0.86, 1.58) | 0.23 |
| Multivariable HR^2^ (95% CI) | 1.00 (ref) | 1.05 (0.76, 1.45) | 0.94 (0.66, 1.35) | 1.10 (0.75, 1.59) | 1.20 (0.76, 1.89) | 0.61 |
| Multivariable HR^3^ (95% CI) | 1.00 (ref) | 1.02 (0.73, 1.42) | 0.90 (0.62, 1.30) | 1.02 (0.69, 1.52) | 1.07 (0.66, 1.73) | 0.99 |
| **Folate** | **Q1** | **Q2** | **Q3** | **Q4** | **Q5** | **P _trend_** |
| Median Intake | 11.72 | 12.58 | 13.17 | 13.78 | 14.72 |  |
| Number of Cases | 73 | 86 | 91 | 84 | 72 |  |
| Age-adjusted HR^1^ (95% CI) | 1.00 (ref) | 1.18 (0.86, 1.61) | 1.26 (0.92, 1.71) | 1.16 (0.85, 1.59) | 1.00 (0.72, 1.38) | 0.94 |
| Multivariable HR^2^ (95% CI) | 1.00 (ref) | 1.17 (0.86, 1.61) | 1.39 (1.01, 1.90) | 1.34 (0.96, 1.87) | 1.40 (0.99, 1.98) | 0.04 |
| Multivariable HR^3^ (95% CI) | 1.00 (ref) | 1.06 (0.76, 1.48) | 1.19 (0.84, 1.69) | 1.12 (0.76, 1.64) | 1.09 (0.70, 1.69) | 0.65 |
| **Vitamin E** | **Q1** | **Q2** | **Q3** | **Q4** | **Q5** | **P _trend_** |
| Median Intake | 1.85 | 2.09 | 2.26 | 2.43 | 2.71 |  |
| Number of Cases | 90 | 98 | 81 | 70 | 65 |  |
| Age-adjusted HR^1^ (95% CI) | 1.00 (ref) | 1.09 (0.82, 1.45) | 0.91 (0.67, 1.23) | 0.79 (0.58, 1.08) | 0.74 (0.54, 1.02) | 0.01 |
| Multivariable HR^2^ (95% CI) | 1.00 (ref) | 1.10 (0.82, 1.48) | 1.00 (0.73, 1.37) | 0.98 (0.71, 1.36) | 0.95 (0.68, 1.33) | 0.60 |
| Multivariable HR^3^ (95% CI) | 1.00 (ref) | 1.08 (0.80, 1.45) | 0.97 (0.70, 1.34) | 0.93 (0.66, 1.31) | 0.84 (0.59, 1.22) | 0.26 |
| **Vitamin D** | **Q1** | **Q2** | **Q3** | **Q4** | **Q5** | **P _trend_** |
| Median Intake | 0.58 | 1.14 | 1.51 | 1.89 | 2.46 |  |
| Number of Cases | 79 | 79 | 99 | 72 | 76 |  |
| Age-adjusted HR^1^ (95% CI) | 1.00 (ref) | 1.01 (0.74, 1.40) | 1.28 (0.93, 1.78) | 0.92 (0.63, 1.34) | 0.90 (0.57, 1.41) | 0.77 |
| Multivariable HR^2^ (95% CI) | 1.00 (ref) | 1.10 (0.79, 1.52) | 1.34 (0.96, 1.88) | 1.02 (0.69, 1.51) | 1.07 (0.67, 1.70) | 0.68 |
| Multivariable HR^3^ (95% CI) | 1.00 (ref) | 1.10 (0.79, 1.53) | 1.37 (0.97, 1.94) | 1.04 (0.69, 1.55) | 1.11 (0.69, 1.78) | 0.59 |
| **Magnesium** | **Q1** | **Q2** | **Q3** | **Q4** | **Q5** | **P _trend_** |
| Median Intake | 10.14 | 10.72 | 11.11 | 11.49 | 12.03 |  |
| Number of Cases | 104 | 81 | 81 | 67 | 70 |  |
| Age-adjusted HR^1^ (95% CI) | 1.00 (ref) | 0.78 (0.59, 1.05) | 0.79 (0.59, 1.06) | 0.66 (0.48, 0.89) | 0.69 (0.51, 0.93) | 0.01 |
| Multivariable HR^2^ (95% CI) | 1.00 (ref) | 0.85 (0.63, 1.14) | 0.99 (0.73, 1.34) | 0.92 (0.66, 1.27) | 1.11 (0.79, 1.56) | 0.51 |
| Multivariable HR^3^ (95% CI) | 1.00 (ref) | 0.77 (0.57, 1.06) | 0.86 (0.62, 1.19) | 0.76 (0.52, 1.09) | 0.80 (0.53, 1.22) | 0.33 |
| **Zinc** | **Q1** | **Q2** | **Q3** | **Q4** | **Q5** | **P _trend_** |
| Median Intake | 2.24 | 2.54 | 2.75 | 2.95 | 3.24 |  |
| Number of Cases | 108 | 92 | 71 | 65 | 70 |  |
| Age-adjusted HR^1^ (95% CI) | 1.00 (ref) | 0.86 (0.65, 1.13) | 0.67 (0.49, 0.90) | 0.62 (0.45, 0.84) | 0.67 (0.50, 0.90) | <0.01 |
| Multivariable HR^2^ (95% CI) | 1.00 (ref) | 0.86 (0.65, 1.15) | 0.81 (0.60, 1.11) | 0.88 (0.63, 1.24) | 1.05 (0.75, 1.50) | 0.94 |
| Multivariable HR^3^ (95% CI) | 1.00 (ref) | 0.85 (0.63, 1.14) | 0.78 (0.56, 1.09) | 0.80 (0.55, 1.17) | 0.96 (0.64, 1.45) | 0.68 |

^1^ Adjusted for entry age ^2^Adjusted for entry age, sex (overall), calories, smoking status, race, education, BMI, and physical activity ^3^Additionally adjusted for

vitamin C, vitamin E, beta-carotene, and folate
