# Supplementary material for: The Association between Selenium and Other Micronutrients and Thyroid Cancer Incidence in the NIH-AARP Diet and Health Study
Source: PLoS One. 2014 Oct 20;9(10):e110886. doi: 10.1371/journal.pone.0110886 (PMC4203851; doi:10.1371/journal.pone.0110886)
Supplement: Table S4 — Hazard Ratios (HRs) and corresponding 95% confidence intervals (CIs) for papillary thyroid cancer by quintile of micronutrient intake among men in The NIH-AARP Diet and Health Study. (DOCX) [file pone.0110886.s004.docx]

**Table S4 – Hazard Ratios (HRs) and corresponding 95% confidence intervals (CIs) for papillary thyroid cancer by quintile of micronutrient intake among men in The NIH-AARP Diet and Health Study:**

| **Selenium** | **Q1** | **Q2** | **Q3** | **Q4** | **Q5** | **P _trend_** |
| --- | --- | --- | --- | --- | --- | --- |
| Median Intake | 7.05 | 7.64 | 8.03 | 8.41 | 8.93 |  |
| Number of Cases | 12 | 14 | 33 | 49 | 56 |  |
| Age-adjusted HR^1^ (95% CI) | 1.00 (ref) | 0.65 (0.30, 1.41) | 1.01 (0.52, 1.96) | 1.17 (0.62, 2.20) | 1.16 (0.62, 2.17) | 0.14 |
| Multivariable HR^2^ (95% CI) | 1.00 (ref) | 0.62 (0.29, 1.34) | 0.87 (0.44, 1.70) | 1.05 (0.56, 1.98) | 1.01 (0.54, 1.89) | 0.30 |
| Multivariable HR^3^ (95% CI) | 1.00 (ref) | 0.76 (0.34, 1.72) | 1.06 (0.51, 2.20) | 1.36 (0.67, 2.75) | 1.32 (0.65, 2.69) | 0.10 |
| **Vitamin C** | **Q1** | **Q2** | **Q3** | **Q4** | **Q5** | **P _trend_** |
| Median Intake | 7 | 8.41 | 9.36 | 10.27 | 11.67 |  |
| Number of Cases | 20 | 24 | 29 | 52 | 37 |  |
| Age-adjusted HR^1^ (95% CI) | 1.00 (ref) | 1.21 (0.67, 2.19) | 1.46 (0.83, 2.59) | 2.58 (1.54, 4.32) | 1.70 (0.98, 2.93) | <0.01 |
| Multivariable HR^2^ (95% CI) | 1.00 (ref) | 1.17 (0.64, 2.12) | 1.27 (0.70, 2.28) | 2.45 (1.46, 4.15) | 1.70 (0.98, 2.98) | 0.01 |
| Multivariable HR^3^ (95% CI) | 1.00 (ref) | 1.19 (0.65, 2.19) | 1.31 (0.71, 2.44) | 2.55 (1.42, 4.59) | 1.88 (0.97, 3.64) | 0.01 |
| **Betacarotene** | **Q1** | **Q2** | **Q3** | **Q4** | **Q5** | **P _trend_** |
| Median Intake | 8.67 | 9.38 | 9.89 | 10.43 | 11.3 |  |
| Number of Cases | 31 | 31 | 33 | 34 | 33 |  |
| Age-adjusted HR^1^ (95% CI) | 1.00 (ref) | 1.01 (0.61, 1.66) | 1.10 (0.68, 1.80) | 1.18 (0.73, 1.93) | 1.24 (0.76, 2.04) | 0.29 |
| Multivariable HR^2^ (95% CI) | 1.00 (ref) | 0.94 (0.57, 1.55) | 1.02 (0.62, 1.68) | 1.14 (0.70, 1.86) | 1.18 (0.71, 1.95) | 0.39 |
| Multivariable HR^3^ (95% CI) | 1.00 (ref) | 0.87 (0.52, 1.46) | 0.89 (0.53, 1.51) | 0.91 (0.53, 1.57) | 0.93 (0.52, 1.67) | 0.86 |
| **Calcium** | **Q1** | **Q2** | **Q3** | **Q4** | **Q5** | **P _trend_** |
| Median Intake | 8.67 | 9.38 | 9.89 | 10.43 | 11.3 |  |
| Number of Cases | 31 | 31 | 33 | 34 | 33 |  |
| Age-adjusted HR^1^ (95% CI) | 1.00 (ref) | 1.01 (0.61, 1.66) | 1.10 (0.68, 1.80) | 1.18 (0.73, 1.93) | 1.24 (0.76, 2.04) | 0.29 |
| Multivariable HR^2^ (95% CI) | 1.00 (ref) | 1.58 (0.86, 2.90) | 1.57 (0.83, 2.95) | 2.01 (1.05, 3.88) | 1.94 (0.90, 4.17) | 0.12 |
| Multivariable HR^3^ (95% CI) | 1.00 (ref) | 1.48 (0.79, 2.80) | 1.45 (0.75, 2.83) | 1.81 (0.90, 3.63) | 1.60 (0.71, 3.59) | 0.36 |
| **Folate** | **Q1** | **Q2** | **Q3** | **Q4** | **Q5** | **P _trend_** |
| Median Intake | 11.72 | 12.58 | 13.17 | 13.78 | 14.72 |  |
| Number of Cases | 12 | 26 | 34 | 41 | 50 |  |
| Age-adjusted HR^1^ (95% CI) | 1.00 (ref) | 1.62 (0.82, 3.22) | 1.81 (0.94, 3.49) | 1.78 (0.94, 3.40) | 1.90 (1.01, 3.57) | 0.08 |
| Multivariable HR^2^ (95% CI) | 1.00 (ref) | 1.60 (0.81, 3.19) | 1.74 (0.90, 3.38) | 1.69 (0.88, 3.26) | 1.97 (1.04, 3.75) | 0.08 |
| Multivariable HR^3^ (95% CI) | 1.00 (ref) | 1.36 (0.68, 2.75) | 1.29 (0.64, 2.62) | 1.18 (0.57, 2.45) | 1.21 (0.55, 2.62) | 0.89 |
| **Vitamin E** | **Q1** | **Q2** | **Q3** | **Q4** | **Q5** | **P _trend_** |
| Median Intake | 1.85 | 2.09 | 2.26 | 2.43 | 2.71 |  |
| Number of Cases | 22 | 29 | 29 | 43 | 41 |  |
| Age-adjusted HR^1^ (95% CI) | 1.00 (ref) | 1.04 (0.60, 1.82) | 0.83 (0.48, 1.45) | 1.06 (0.64, 1.78) | 0.94 (0.56, 1.57) | 0.86 |
| Multivariable HR^2^ (95% CI) | 1.00 (ref) | 1.03 (0.59, 1.80) | 0.79 (0.45, 1.40) | 1.03 (0.61, 1.74) | 0.93 (0.55, 1.59) | 0.02 |
| Multivariable HR^3^ (95% CI) | 1.00 (ref) | 0.96 (0.54, 1.72) | 0.72 (0.40, 1.30) | 0.90 (0.51, 1.58) | 0.78 (0.44, 1.40) | 0.44 |
| **Vitamin D** | **Q1** | **Q2** | **Q3** | **Q4** | **Q5** | **P _trend_** |
| Median Intake | 0.58 | 1.14 | 1.51 | 1.89 | 2.46 |  |
| Number of Cases | 22 | 32 | 38 | 35 | 37 |  |
| Age-adjusted HR^1^ (95% CI) | 1.00 (ref) | 1.05 (0.60, 1.82) | 1.00 (0.57, 1.75) | 0.78 (0.42, 1.42) | 0.73 (0.36, 1.48) | 0.22 |
| Multivariable HR^2^ (95% CI) | 1.00 (ref) | 1.05 (0.60, 1.83) | 0.97 (0.55, 1.71) | 0.73 (0.40, 1.35) | 0.70 (0.34, 1.43) | 0.17 |
| Multivariable HR^3^ (95% CI) | 1.00 (ref) | 1.10 (0.71, 1.97) | 1.06 (0.58, 1.91) | 0.81 (0.43, 1.53) | 0.79 (0.38, 1.67) | 0.31 |
| **Magnesium** | **Q1** | **Q2** | **Q3** | **Q4** | **Q5** | **P _trend_** |
| Median Intake | 10.14 | 10.72 | 11.11 | 11.49 | 12.03 |  |
| Number of Cases | 15 | 23 | 32 | 39 | 54 |  |
| Age-adjusted HR^1^ (95% CI) | 1.00 (ref) | 1.01 (0.53, 1.94) | 1.09 (0.59, 2.02) | 1.10 (0.61, 1.99) | 1.30 (0.73, 2.3) | 0.25 |
| Multivariable HR^2^ (95% CI) | 1.00 (ref) | 1.01 (0.52, 1.97) | 1.13 (0.60, 2.12) | 1.10 (0.59, 2.03) | 1.35 (0.75 2.45) | 0.20 |
| Multivariable HR^3^ (95% CI) | 1.00 (ref) | 1.00 (0.50, 2.02) | 1.06 (0.54, 2.08) | 0.97 (0.49, 1.94) | 1.06 (0.52, 2.17) | 0.83 |
| **Zinc** | **Q1** | **Q2** | **Q3** | **Q4** | **Q5** | **P _trend_** |
| Median Intake | 2.24 | 2.54 | 2.75 | 2.95 | 3.24 |  |
| Number of Cases | 15 | 14 | 29 | 45 | 61 |  |
| Age-adjusted HR^1^ (95% CI) | 1.00 (ref) | 0.55 (0.27, 1.15) | 0.74 (0.40, 1.39) | 0.87 (0.49, 1.57) | 1.06 (0.60, 1.87) | 0.12 |
| Multivariable HR^2^ (95% CI) | 1.00 (ref) | 0.47 (0.22, 0.99) | 0.67 (0.36, 1.25) | 0.75 (0.42, 1.36) | 0.93 (0.52, 1.64) | 0.23 |
| Multivariable HR^3^ (95% CI) | 1.00 (ref) | 0.49 (0.22, 1.08) | 0.67 (0.33, 1.35) | 0.74 (0.37, 1.48) | 0.87 (0.42, 1.80) | 0.36 |

^1^ Adjusted for entry age ^2^Adjusted for entry age, sex (overall), calories, smoking status, race, education, BMI, and physical activity ^3^Additionally adjusted for

vitamin C, vitamin E, beta-carotene, and folate
