# Supplementary material for: The Association between Selenium and Other Micronutrients and Thyroid Cancer Incidence in the NIH-AARP Diet and Health Study
Source: PLoS One. 2014 Oct 20;9(10):e110886. doi: 10.1371/journal.pone.0110886 (PMC4203851; doi:10.1371/journal.pone.0110886)
Supplement: Table S5 — Hazard Ratios (HRs) and corresponding 95% confidence intervals (CIs) for papillary thyroid cancer by quintile of micronutrient intake among women in The NIH-AARP Diet and Health Study. (DOCX) [file pone.0110886.s005.docx]

**Table S5 – Hazard Ratios (HRs) and corresponding 95% confidence intervals (CIs) for papillary thyroid cancer by quintile of micronutrient intake among women in The NIH-AARP Diet and Health Study:**

| **Selenium** | **Q1** | **Q2** | **Q3** | **Q4** | **Q5** | **P _trend_** |
| --- | --- | --- | --- | --- | --- | --- |
| Median Intake | 7.05 | 7.64 | 8.03 | 8.41 | 8.93 |  |
| Number of Cases | 83 | 77 | 49 | 21 | 12 |  |
| Age-adjusted HR^1^ (95% CI) | 1.00 (ref) | 1.20 (0.88, 1.64) | 1.19 (0.84, 1.70) | 0.96 (0.60, 1.55) | 1.29 (0.71, 2.37) | 0.45 |
| Multivariable HR^2^ (95% CI) | 1.00 (ref) | 1.14 (0.83, 1.57) | 1.09 (0.76, 1.58) | 0.92 (0.56, 1.50) | 1.29 (0.70, 2.38) | 0.65 |
| Multivariable HR^3^ (95% CI) | 1.00 (ref) | 1.21 (0.87, 1.67) | 1.17 (0.80, 1.72) | 0.99 (0.60, 1.65) | 1.29 (0.68, 2.46) | 0.48 |
| **Vitamin C** | **Q1** | **Q2** | **Q3** | **Q4** | **Q5** | **P _trend_** |
| Median Intake | 7 | 8.41 | 9.36 | 10.27 | 11.67 |  |
| Number of Cases | 20 | 24 | 29 | 52 | 37 |  |
| Age-adjusted HR^1^ (95% CI) | 1.00 (ref) | 1.21 (0.67, 2.19) | 1.46 (0.83, 2.59) | 2.58 (1.54, 4.32) | 1.70 (0.98, 2.93) | <0.01 |
| Multivariable HR^2^ (95% CI) | 1.00 (ref) | 1.17 (0.64, 2.12) | 1.27 (0.70, 2.28) | 2.45 (1.46, 4.15) | 1.70 (0.98, 2.98) | 0.01 |
| Multivariable HR^3^ (95% CI) | 1.00 (ref) | 1.19 (0.65, 2.19) | 1.31 (0.71, 2.44) | 2.55 (1.42, 4.59) | 1.88 (0.97, 3.64) | 0.01 |
| **Betacarotene** | **Q1** | **Q2** | **Q3** | **Q4** | **Q5** | **P _trend_** |
| Median Intake | 8.67 | 9.38 | 9.89 | 10.43 | 11.3 |  |
| Number of Cases | 46 | 44 | 44 | 50 | 57 |  |
| Age-adjusted HR^1^ (95% CI) | 1.00 (ref) | 0.94 (0.62, 1.42) | 0.89 (0.59, 1.35) | 0.95 (0.64, 1.42) | 0.98 (0.66, 1.44) | 0.99 |
| Multivariable HR^2^ (95% CI) | 1.00 (ref) | 0.94 (0.62, 1.43) | 0.81 (0.53, 1.25) | 0.94 (0.62, 1.42) | 0.96 (0.64, 1.45) | 0.88 |
| Multivariable HR^3^ (95% CI) | 1.00 (ref) | 0.85 (0.56, 1.31) | 0.72 (0.46, 1.12) | 0.79 (0.51, 1.24) | 0.76 (0.47, 1.22) | 0.25 |
| **Calcium** | **Q1** | **Q2** | **Q3** | **Q4** | **Q5** | **P _trend_** |
| Median Intake | 8.67 | 9.38 | 9.89 | 10.43 | 11.3 |  |
| Number of Cases | 46 | 44 | 44 | 50 | 57 |  |
| Age-adjusted HR^1^ (95% CI) | 1.00 (ref) | 0.94 (0.62, 1.42) | 0.89 (0.59, 1.35) | 0.95 (0.64, 1.42) | 0.98 (0.66, 1.44) | 0.99 |
| Multivariable HR^2^ (95% CI) | 1.00 (ref) | 0.88 (0.59, 1.30) | 0.73 (0.47, 1.15) | 0.78 (0.48, 1.26) | 0.97 (0.54, 1.73) | 0.53 |
| Multivariable HR^3^ (95% CI) | 1.00 (ref) | 0.86 (0.58, 1.29) | 0.71 (0.44, 1.12) | 0.74 (0.45, 1.23) | 0.89 (0.48, 1.66) | 0.42 |
| **Folate** | **Q1** | **Q2** | **Q3** | **Q4** | **Q5** | **P _trend_** |
| Median Intake | 11.72 | 12.58 | 13.17 | 13.78 | 14.72 |  |
| Number of Cases | 61 | 60 | 56 | 43 | 22 |  |
| Age-adjusted HR^1^ (95% CI) | 1.00 (ref) | 1.22 (0.85, 1.74) | 1.43 (0.99, 2.05) | 1.49 (1.01, 2.20) | 1.14 (0.70, 1.86) | 0.13 |
| Multivariable HR^2^ (95% CI) | 1.00 (ref) | 1.07 (0.74, 1.54) | 1.33 (0.92, 1.92) | 1.31 (0.87, 1.96) | 1.15 (0.70, 1.88) | 0.19 |
| Multivariable HR^3^ (95% CI) | 1.00 (ref) | 1.01 (0.68, 1.49) | 1.24 (0.81,1.88) | 1.20 (0.74, 1.95) | 1.05 (0.57, 1.93) | 0.45 |
| **Vitamin E** | **Q1** | **Q2** | **Q3** | **Q4** | **Q5** | **P _trend_** |
| Median Intake | 1.85 | 2.09 | 2.26 | 2.43 | 2.71 |  |
| Number of Cases | 68 | 69 | 52 | 27 | 24 |  |
| Age-adjusted HR^1^ (95% CI) | 1.00 (ref) | 1.21 (0.87, 1.70) | 1.21 (0.84, 1.74) | 0.85 (0.54, 1.33) | 0.96 (0.60, 1.53) | 0.67 |
| Multivariable HR^2^ (95% CI) | 1.00 (ref) | 1.13 (0.80, 1.60) | 1.16 (0.80, 1.68) | 0.88 (0.56, 1.37) | 0.91 (0.56, 1.48) | 0.61 |
| Multivariable HR^3^ (95% CI) | 1.00 (ref) | 1.13 (0.80, 1.60) | 1.15 (0.79, 1.69) | 0.86 (0.54, 1.37) | 0.80 (0.48, 1.35) | 0.38 |
| **Vitamin D** | **Q1** | **Q2** | **Q3** | **Q4** | **Q5** | **P _trend_** |
| Median Intake | 0.58 | 1.14 | 1.51 | 1.89 | 2.46 |  |
| Number of Cases | 57 | 47 | 61 | 37 | 39 |  |
| Age-adjusted HR^1^ (95% CI) | 1.00 (ref) | 1.07 (0.72, 1.60) | 1.72 (1.15, 2.57) | 1.25 (0.76, 2.04) | 1.40 (0.77, 2.54) | 0.10 |
| Multivariable HR^2^ (95% CI) | 1.00 (ref) | 1.11 (0.74, 1.67) | 1.63 (1.07, 2.48) | 1.24 (0.75, 2.07) | 1.44 (0.78, 2.65) | 0.10 |
| Multivariable HR^3^ (95% CI) | 1.00 (ref) | 1.09 (0.72, 1.64) | 1.63 (1.06, 2.50) | 1.21 (0.72, 2.05) | 1.43 (0.76, 2.69) | 0.13 |
| **Magnesium** | **Q1** | **Q2** | **Q3** | **Q4** | **Q5** | **P _trend_** |
| Median Intake | 10.14 | 10.72 | 11.11 | 11.49 | 12.03 |  |
| Number of Cases | 89 | 58 | 49 | 28 | 16 |  |
| Age-adjusted HR^1^ (95% CI) | 1.00 (ref) | 0.84 (0.61, 1.18) | 0.96 (0.68, 1.36) | 0.81 (0.53, 1.24) | 0.87 (0.51, 1.48) | 0.41 |
| Multivariable HR^2^ (95% CI) | 1.00 (ref) | 0.82 (0.58, 1.16) | 1.00 (0.70, 1.43) | 0.88 (0.57, 1.36) | 0.98 (0.57, 1.68) | 0.85 |
| Multivariable HR^3^ (95% CI) | 1.00 (ref) | 0.74 (0.52, 1.06) | 0.84 (0.57, 1.25) | 0.71 (0.44, 1.15) | 0.66 (0.35, 1.26) | 0.15 |
| **Zinc** | **Q1** | **Q2** | **Q3** | **Q4** | **Q5** | **P _trend_** |
| Median Intake | 2.24 | 2.54 | 2.75 | 2.95 | 3.24 |  |
| Number of Cases | 93 | 78 | 42 | 20 | 9 |  |
| Age-adjusted HR^1^ (95% CI) | 1.00 (ref) | 1.06 (0.78, 1.43) | 0.89 (0.62, 1.28) | 0.87 (0.54, 1.41) | 0.82 (0.42, 1.63) | 0.4 |
| Multivariable HR^2^ (95% CI) | 1.00 (ref) | 0.98 (0.72, 1.33) | 0.84 (0.58, 1.22) | 0.85 (0.52, 1.38) | 0.81 (0.41, 1.61) | 0.35 |
| Multivariable HR^3^ (95% CI) | 1.00 (ref) | 0.95 (0.69, 1.30) | 0.79 (0.53, 1.17) | 0.70 (0.41, 1.20) | 0.75 (0.36, 1.57) | 0.15 |

^1^ Adjusted for entry age ^2^Adjusted for entry age, sex (overall), calories, smoking status, race, education, BMI, and physical activity ^3^Additionally adjusted for

vitamin C, vitamin E, beta-carotene, and folate
