# Supplementary material for: The Association between Selenium and Other Micronutrients and Thyroid Cancer Incidence in the NIH-AARP Diet and Health Study
Source: PLoS One. 2014 Oct 20;9(10):e110886. doi: 10.1371/journal.pone.0110886 (PMC4203851; doi:10.1371/journal.pone.0110886)
Supplement: Table S6 — Hazard Ratios (HRs) and corresponding 95% confidence intervals (CIs) for total thyroid cancer by quintile of micronutrient intake among men in The NIH-AARP Diet and Health Study. (DOCX) [file pone.0110886.s006.docx]

**Table S6 – Hazard Ratios (HRs) and corresponding 95% confidence intervals (CIs) for total thyroid cancer by quintile of micronutrient intake among men in The NIH-AARP Diet and Health Study:**

| **Selenium** | **Q1** | **Q2** | **Q3** | **Q4** | **Q5** | **P _trend_** |
| --- | --- | --- | --- | --- | --- | --- |
| Median Intake | 7.05 | 7.64 | 8.03 | 8.41 | 8.93 |  |
| Number of Cases | 19 | 25 | 49 | 68 | 96 |  |
| Age-adjusted HR^1^ (95% CI) | 1.00 (ref) | 0.97 (0.72, 1.29) | 0.88 (0.65, 1.18) | 0.76 (0.55, 1.03) | 0.73 (0.54, 1.00) | 0.02 |
| Multivariable HR^2^ (95% CI) | 1.00 (ref) | 0.69 (0.38, 1.25) | 0.82 (0.48, 1.40) | 0.91 (0.55, 1.53) | 1.08 (0.66, 1.77) | 0.13 |
| Multivariable HR^3^ (95% CI) | 1.00 (ref) | 0.74 (0.39, 1.38) | 0.90 (0.51, 1.59) | 1.04 (0.60, 1.79) | 1.23 (0.71, 2.12) | 0.05 |
| **Vitamin C** | **Q1** | **Q2** | **Q3** | **Q4** | **Q5** | **P _trend_** |
| Median Intake | 7 | 8.41 | 9.36 | 10.28 | 11.67 |  |
| Number of Cases | 36 | 43 | 46 | 67 | 63 |  |
| Age-adjusted HR^1^ (95% CI) | 1.00 (ref) | 1.19 (0.77, 1.86) | 1.27 (0.82, 1.96) | 1.80 (1.20, 2.70) | 1.57 (1.20, 2.70) | 0.01 |
| Multivariable HR^2^ (95% CI) | 1.00 (ref) | 1.11 (0.71, 1.73) | 1.11 (0.71, 1.74) | 1.67 (1.10, 2.52) | 1.50 (0.98, 2.28) | 0.01 |
| Multivariable HR^3^ (95% CI) | 1.00 (ref) | 1.09 (0.69, 1.72) | 1.10 (0.69, 1.77) | 1.61 (1.01, 2.57) | 1.55 (0.93, 2.57) | 0.03 |
| **Betacarotene** | **Q1** | **Q2** | **Q3** | **Q4** | **Q5** | **P _trend_** |
| Median Intake | 8.67 | 9.38 | 9.89 | 10.43 | 11.3 |  |
| Number of Cases | 47 | 49 | 50 | 57 | 52 |  |
| Age-adjusted HR^1^ (95% CI) | 1.00 (ref) | 1.04 (0.70, 1.55) | 1.09 (0.73, 1.62) | 1.29 (0.87, 1.89) | 1.27 (0.86, 1.89) | 0.13 |
| Multivariable HR^2^ (95% CI) | 1.00 (ref) | 0.97 (0.65, 1.46) | 1.01 (0.67, 1.51) | 1.18 (0.79, 1.75) | 1.20 (0.80, 1.01) | 0.25 |
| Multivariable HR^3^ (95% CI) | 1.00 (ref) | 0.90 (0.60, 1.37) | 0.88 (0.58, 1.35) | 0.96 (0.63, 1.47) | 0.93 (0.58, 1.49) | 0.88 |
| **Calcium** | **Q1** | **Q2** | **Q3** | **Q4** | **Q5** | **P _trend_** |
| Median Intake | 8.67 | 9.38 | 9.89 | 10.43 | 11.3 |  |
| Number of Cases | 47 | 49 | 50 | 57 | 52 |  |
| Age-adjusted HR^1^ (95% CI) | 1.00 (ref) | 1.04 (0.70, 1.55) | 1.09 (0.73, 1.62) | 1.29 (0.87, 1.89) | 1.27 (0.86, 1.89) | 0.13 |
| Multivariable HR^2^ (95% CI) | 1.00 (ref) | 1.24 (0.77, 2.02) | 1.25 (0.76, 2.06) | 1.55 (0.93, 2.59) | 1.39 (0.76, 2.54) | 0.33 |
| Multivariable HR^3^ (95% CI) | 1.00 (ref) | 1.16 (0.70, 1.90) | 1.14 (0.68, 1.93) | 1.39 (0.81, 2.38) | 1.15 (0.61, 2.17) | 0.76 |
| **Folate** | **Q1** | **Q2** | **Q3** | **Q4** | **Q5** | **P _trend_** |
| Median Intake | 11.72 | 12.58 | 13.17 | 13.78 | 14.72 |  |
| Number of Cases | 25 | 36 | 51 | 58 | 87 |  |
| Age-adjusted HR^1^ (95% CI) | 1.00 (ref) | 1.07 (0.64, 1.78) | 1.25 (0.77, 2.01) | 1.19 (0.74, 1.90) | 1.55 (0.99, 2.42) | 0.02 |
| Multivariable HR^2^ (95% CI) | 1.00 (ref) | 1.04 (0.63, 1.74) | 1.19 (0.73, 1.93) | 1.11 (0.69, 1.80) | 1.52 (0.96, 2.40) | 0.04 |
| Multivariable HR^3^ (95% CI) | 1.00 (ref) | 0.90 (0.53, 1.53) | 0.92 (0.55, 1.56) | 0.83 (0.48, 1.42) | 1.02 (0.58, 1.81) | 0.86 |
| **Vitamin E** | **Q1** | **Q2** | **Q3** | **Q4** | **Q5** | **P _trend_** |
| Median Intake | 1.85 | 2.09 | 2.26 | 2.43 | 2.71 |  |
| Number of Cases | 29 | 46 | 48 | 67 | 66 |  |
| Age-adjusted HR^1^ (95% CI) | 1.00 (ref) | 1.25 (0.79, 1.99) | 1.04 (0.65, 1.65) | 1.24 (0.80, 1.92) | 1.12 (0.72, 1.74) | 0.75 |
| Multivariable HR^2^ (95% CI) | 1.00 (ref) | 1.18 (0.74, 1.90) | 0.98 (0.61, 1.57) | 1.17 (0.75, 1.82) | 1.11 (0.71, 1.73) | 0.73 |
| Multivariable HR^3^ (95% CI) | 1.00 (ref) | 1.11 (0.68, 1.80) | 0.89 (0.55, 1.45) | 1.03 (0.64, 1.66) | 0.95 (0.59, 1.54) | 0.73 |
| **Vitamin D** | **Q1** | **Q2** | **Q3** | **Q4** | **Q5** | **P _trend_** |
| Median Intake | 0.58 | 1.14 | 1.51 | 1.89 | 2.46 |  |
| Number of Cases | 31 | 46 | 56 | 64 | 60 |  |
| Age-adjusted HR^1^ (95% CI) | 1.00 (ref) | 1.11 (0.70, 1.76) | 1.11 (0.69, 1.77) | 1.07 (0.66, 1.75) | 0.95 (0.54, 1.68) | 0.75 |
| Multivariable HR^2^ (95% CI) | 1.00 (ref) | 1.13 (0.71, 1.82) | 1.11 (0.69, 1.80) | 1.07 (0.65, 1.77) | 0.94 (0.52, 1.67) | 0.71 |
| Multivariable HR^3^ (95% CI) | 1.00 (ref) | 1.16 (0.71, 1.88) | 1.16 (0.71, 1.91) | 1.11 (0.66, 1.87) | 1.02 (0.56, 1.86) | 0.92 |
| **Magnesium** | **Q1** | **Q2** | **Q3** | **Q4** | **Q5** | **P _trend_** |
| Median Intake | 10.14 | 10.72 | 11.11 | 11.49 | 12.03 |  |
| Number of Cases | 21 | 38 | 51 | 67 | 79 |  |
| Age-adjusted HR^1^ (95% CI) | 1.00 (ref) | 1.19 (0.70, 2.02) | 1.23 (0.74, 2.05) | 1.33 (0.82, 2.18) | 1.34 (0.83, 2.17) | 0.21 |
| Multivariable HR^2^ (95% CI) | 1.00 (ref) | 1.17 (0.68, 2.02) | 1.23 (0.73, 2.06) | 1.29 (0.78, 2.14) | 1.30 (0.79, 2.13) | 0.30 |
| Multivariable HR^3^ (95% CI) | 1.00 (ref) | 1.13 (0.64, 1.98) | 1.11 (0.64, 1.93) | 1.09 (0.62, 1.91) | 1.00 (0.56, 1.80) | 0.81 |
| **Zinc** | **Q1** | **Q2** | **Q3** | **Q4** | **Q5** | **P _trend_** |
| Median Intake | 2.24 | 2.54 | 2.75 | 2.95 | 3.24 |  |
| Number of Cases | 17 | 31 | 45 | 77 | 87 |  |
| Age-adjusted HR^1^ (95% CI) | 1.00 (ref) | 1.08 (0.60, 1.95) | 1.02 (0.58, 1.78) | 1.32 (0.78, 2.23) | 1.34 (0.80, 2.25) | 0.10 |
| Multivariable HR^2^ (95% CI) | 1.00 (ref) | 0.95 (0.52, 1.72) | 0.90 (0.52, 1.58) | 1.10 (0.65, 1.88) | 1.15 (0.68, 1.95) | 0.26 |
| Multivariable HR^3^ (95% CI) | 1.00 (ref) | 0.95 (0.50, 1.79) | 0.85 (0.46, 1.58) | 0.99 (0.54, 1.83) | 0.98 (0.52, 1.84) | 0.80 |

^1^ Adjusted for entry age ^2^Adjusted for entry age, sex (overall), calories, smoking status, race, education, BMI, and physical activity ^3^Additionally adjusted for

vitamin C, vitamin E, beta-carotene, and folate

**Table S2 – Hazard Ratios (HRs) and corresponding 95% confidence intervals (CIs) for total thyroid cancer by quintile of micronutrient intake among women in The NIH-AARP Diet and Health Study:**

| **Selenium** | **Q1** | **Q2** | **Q3** | **Q4** | **Q5** | **P _trend_** |
| --- | --- | --- | --- | --- | --- | --- |
| Median Intake | 7.05 | 7.64 | 8.03 | 8.41 | 8.93 |  |
| Number of Cases | 119 | 106 | 66 | 29 | 15 |  |
| Age-adjusted HR^1^ (95% CI) | 1.00 (ref) | 0.89 (0.50, 1.58) | 0.90 (0.51, 1.59) | 0.79 (0.44, 1.43) | 1.05 (0.60, 1.83) | 0.67 |
| Multivariable HR^2^ (95% CI) | 1.00 (ref) | 1.11 (0.85, 1.45) | 1.06 (0.78, 1.44) | 0.90 (0.60, 1.37) | 1.12 (0.65, 1.93) | 0.90 |
| Multivariable HR^3^ (95% CI) | 1.00 (ref) | 1.17 (0.89, 1.54) | 1.14 (0.82, 1.56) | 0.98 (0.64, 1.50) | 1.14 (0.65, 2.02) | 0.62 |
| **Vitamin C** | **Q1** | **Q2** | **Q3** | **Q4** | **Q5** | **P _trend_** |
| Median Intake | 7 | 8.41 | 9.36 | 10.28 | 11.67 |  |
| Number of Cases | 58 | 62 | 68 | 80 | 66 |  |
| Age-adjusted HR^1^ (95% CI) | 1.00 (ref) | 1.04 (0.73, 1.49) | 1.14 (0.80, 1.62) | 1.39 (0.99, 1.95) | 1.29 (0.91, 1.84) | 0.05 |
| Multivariable HR^2^ (95% CI) | 1.00 (ref) | 0.98 (0.68, 1.41) | 1.05 (0.73, 1.50) | 1.28 (0.90, 1.81) | 1.25 (0.87, 1.80) | 0.09 |
| Multivariable HR^3^ (95% CI) | 1.00 (ref) | 0.99 (0.68, 1.44) | 1.11 (0.76, 1.62) | 1.37 (0.93, 2.03) | 1.40 (0.90, 2.19) | 0.06 |
| **Betacarotene** | **Q1** | **Q2** | **Q3** | **Q4** | **Q5** | **P _trend_** |
| Median Intake | 8.67 | 9.38 | 9.89 | 10.43 | 11.3 |  |
| Number of Cases | 64 | 67 | 57 | 66 | 80 |  |
| Age-adjusted HR^1^ (95% CI) | 1.00 (ref) | 1.02 (0.72, 1.43) | 0.82 (0.57, 1.17) | 0.89 (0.63, 1.26) | 0.97 (0.70, 1.34) | 0.74 |
| Multivariable HR^2^ (95% CI) | 1.00 (ref) | 1.02 (0.72, 1.44) | 0.78 (0.54, 1.12) | 0.90 (0.63, 1.28) | 1.00 (0.71, 1.41) | 0.82 |
| Multivariable HR^3^ (95% CI) | 1.00 (ref) | 0.95 (0.67, 1.36) | 0.70 (0.48, 1.03) | 0.81 (0.56, 1.19) | 0.87 (0.59, 1.30) | 0.36 |
| **Calcium** | **Q1** | **Q2** | **Q3** | **Q4** | **Q5** | **P _trend_** |
| Median Intake | 8.67 | 9.38 | 9.89 | 10.43 | 11.3 |  |
| Number of Cases | 64 | 67 | 57 | 66 | 80 |  |
| Age-adjusted HR^1^ (95% CI) | 1.00 (ref) | 1.02 (0.72, 1.43) | 0.82 (0.57, 1.17) | 0.89 (0.63, 1.26) | 0.97 (0.70, 1.34) | 0.74 |
| Multivariable HR^2^ (95% CI) | 1.00 (ref) | 0.82 (0.59, 1.15) | 0.79 (0.55, 1.15) | 1.00 (0.67, 1.48) | 1.00 (0.60, 1.65) | 0.87 |
| Multivariable HR^3^ (95% CI) | 1.00 (ref) | 0.83 (0.59, 1.17) | 0.79 (0.54, 1.17) | 1.00 (0.66, 1.52) | 0.98 (0.57, 1.67) | 0.83 |
| **Folate** | **Q1** | **Q2** | **Q3** | **Q4** | **Q5** | **P _trend_** |
| Median Intake | 11.72 | 12.58 | 13.17 | 13.78 | 14.72 |  |
| Number of Cases | 96 | 79 | 78 | 52 | 30 |  |
| Age-adjusted HR^1^ (95% CI) | 1.00 (ref) | 1.01 (0.75, 1.36) | 1.25 (0.93, 1.69) | 1.13 (0.81, 1.59) | 0.99 (0.65, 1.48) | 0.55 |
| Multivariable HR^2^ (95% CI) | 1.00 (ref) | 0.92 (0.68, 1.25) | 1.21 (0.90, 1.64) | 1.05 (0.74, 1.49) | 1.02 (0.67, 1.55) | 0.49 |
| Multivariable HR^3^ (95% CI) | 1.00 (ref) | 0.86 (0.62, 1.19) | 1.11 (0.79, 1.58) | 0.94 (0.62, 1.43) | 0.89 (0.52, 1.47) | 0.98 |
| **Vitamin E** | **Q1** | **Q2** | **Q3** | **Q4** | **Q5** | **P _trend_** |
| Median Intake | 1.85 | 2.09 | 2.26 | 2.43 | 2.71 |  |
| Number of Cases | 29 | 46 | 48 | 67 | 66 |  |
| Age-adjusted HR^1^ (95% CI) | 1.00 (ref) | 1.25 (0.79, 1.99) | 1.04 (0.65, 1.65) | 1.24 (0.80, 1.92) | 1.12 (0.72, 1.74) | 0.75 |
| Multivariable HR^2^ (95% CI) | 1.00 (ref) | 1.18 (0.74, 1.90) | 0.98 (0.61, 1.57) | 1.17 (0.75, 1.82) | 1.11 (0.71, 1.73) | 0.73 |
| Multivariable HR^3^ (95% CI) | 1.00 (ref) | 1.11 (0.68, 1.80) | 0.89 (0.55, 1.45) | 1.03 (0.64, 1.66) | 0.95 (0.59, 1.54) | 0.73 |
| **Vitamin D** | **Q1** | **Q2** | **Q3** | **Q4** | **Q5** | **P _trend_** |
| Median Intake | 0.58 | 1.14 | 1.51 | 1.89 | 2.46 |  |
| Number of Cases | 83 | 70 | 79 | 53 | 49 |  |
| Age-adjusted HR^1^ (95% CI) | 1.00 (ref) | 1.09 (0.79, 1.51) | 1.45 (1.03, 2.04) | 1.09 (0.72, 1.65) | 1.13 (0.68, 1.89) | 0.34 |
| Multivariable HR^2^ (95% CI) | 1.00 (ref) | 1.09 (0.78, 1.52) | 1.36 (0.95, 1.94) | 1.07 (0.70, 1.63) | 1.10 (0.65, 1.85) | 0.45 |
| Multivariable HR^3^ (95% CI) | 1.00 (ref) | 1.06 (0.76, 1.49) | 1.34 (0.93, 1.93) | 1.03 (0.67, 1.60) | 1.05 (0.61, 1.81) | 0.57 |
| **Magnesium** | **Q1** | **Q2** | **Q3** | **Q4** | **Q5** | **P _trend_** |
| Median Intake | 10.14 | 10.72 | 11.11 | 11.49 | 12.03 |  |
| Number of Cases | 124 | 77 | 65 | 46 | 21 |  |
| Age-adjusted HR^1^ (95% CI) | 1.00 (ref) | 0.80 (0.60, 1.06) | 0.91 (0.67, 1.22) | 0.95 (0.67, 1.33) | 0.81 (0.51, 1.28) | 0.41 |
| Multivariable HR^2^ (95% CI) | 1.00 (ref) | 0.79 (0.59, 1.05) | 0.92 (0.68, 1.25) | 1.01 (0.72, 1.43) | 0.89 (0.55, 1.42) | 0.76 |
| Multivariable HR^3^ (95% CI) | 1.00 (ref) | 0.74 (0.55, 1.01) | 0.85 (0.60, 1.18) | 0.89 (0.60, 1.32) | 0.71 (0.42, 1.25) | 0.26 |
| **Zinc** | **Q1** | **Q2** | **Q3** | **Q4** | **Q5** | **P _trend_** |
| Median Intake | 2.24 | 2.54 | 2.75 | 2.95 | 3.24 |  |
| Number of Cases | 129 | 107 | 61 | 27 | 11 |  |
| Age-adjusted HR^1^ (95% CI) | 1.00 (ref) | 1.04 (0.81, 1.35) | 0.93 (0.69, 1.26) | 0.85 (0.56, 1.29) | 0.72 (0.39, 1.34) | 0.25 |
| Multivariable HR^2^ (95% CI) | 1.00 (ref) | 0.98 (0.75, 1.28) | 0.87 (0.64, 1.19) | 0.82 (0.54, 1.25) | 0.70 (0.38, 1.30) | 0.75 |
| Multivariable HR^3^ (95% CI) | 1.00 (ref) | 0.98 (0.74, 1.28) | 0.86 (0.61, 1.19) | 0.74 (0.47, 1.17) | 0.70 (0.36, 1.36) | 0.11 |

^1^ Adjusted for entry age ^2^Adjusted for entry age, sex (overall), calories, smoking status, race, education, BMI, and physical activity ^3^Additionally adjusted for

vitamin C, vitamin E, beta-carotene, and folate

**Table S3 – Hazard Ratios (HRs) and corresponding 95% confidence intervals (CIs) for papillary thyroid cancer by quintile of micronutrient intake among men and women combined in The NIH-AARP Diet and Health Study:**

| **Selenium** | **Q1** | **Q2** | **Q3** | **Q4** | **Q5** | **P _trend_** |
| --- | --- | --- | --- | --- | --- | --- |
| Median Intake | 7.05 | 7.64 | 8.03 | 8.41 | 8.93 |  |
| Number of Cases | 95 | 91 | 82 | 70 | 68 |  |
| Age-adjusted HR^1^ (95% CI) | 1.00 (ref) | 0.97 (0.72, 1.29) | 0.88 (0.65, 1.18) | 0.76, 0.55, 1.03) | 0.73 (0.54, 1.00) | 0.02 |
| Multivariable HR^2^ (95% CI) | 1.00 (ref) | 1.02 (0.76, 1.37) | 1.04 (0.76, 1.43) | 1.11 (0.79, 1.56) | 1.20 (0.83, 1.72) | 0.32 |
| Multivariable HR^3^ (95% CI) | 1.00 (ref) | 1.11 (0.82, 1.50) | 1.14 (0.83, 1.58) | 1.26 (0.89, 1.79) | 1.35 (0.92, 1.98) | 0.11 |
| **Vitamin C** | **Q1** | **Q2** | **Q3** | **Q4** | **Q5** | **P _trend_** |
| Median Intake | 7 | 8.41 | 9.36 | 10.27 | 11.67 |  |
| Number of Cases | 20 | 24 | 29 | 52 | 37 |  |
| Age-adjusted HR^1^ (95% CI) | 1.00 (ref) | 1.21 (0.67, 2.19) | 1.46 (0.83, 2.59) | 2.58 (1.54, 4.32) | 1.7 (0.98, 2.93) | <0.01 |
| Multivariable HR^2^ (95% CI) | 1.00 (ref) | 1.10 (0.77, 1.58) | 1.35 (0.96, 1.92) | 1.93 (1.39, 2.69) | 1.47 (1.03, 2.09) | <0.01 |
| Multivariable HR^3^ (95% CI) | 1.00 (ref) | 1.10 (0.76, 1.59) | 1.41 (0.98, 2.04) | 2.02 (1.40, 2.91) | 1.60 (1.05, 2.43) | <0.01 |
| **Betacarotene** | **Q1** | **Q2** | **Q3** | **Q4** | **Q5** | **P _trend_** |
| Median Intake | 8.67 | 9.38 | 9.89 | 10.43 | 11.3 |  |
| Number of Cases | 77 | 75 | 77 | 84 | 90 |  |
| Age-adjusted HR^1^ (95% CI) | 1.00 (ref) | 0.97 (0.71, 1.34) | 1.00 (0.73, 1.37) | 1.09 (0.80, 1.47) | 1.16 (0.86, 1.58) | 0.23 |
| Multivariable HR^2^ (95% CI) | 1.00 (ref) | 0.94 (0.68, 1.30) | 0.89 (0.65, 1.24) | 1.01 (0.75, 1.39) | 1.04 (0.76, 1.43) | 0.70 |
| Multivariable HR^3^ (95% CI) | 1.00 (ref) | 0.86 (0.62, 1.20) | 0.79 (0.56, 1.10) | 0.84 (0.60, 1.18) | 0.82 (0.57, 1.18) | 0.31 |
| **Calcium** | **Q1** | **Q2** | **Q3** | **Q4** | **Q5** | **P _trend_** |
| Median Intake | 8.67 | 9.38 | 9.89 | 10.43 | 11.3 |  |
| Number of Cases | 77 | 75 | 77 | 84 | 90 |  |
| Age-adjusted HR^1^ (95% CI) | 1.00 (ref) | 0.97 (0.71, 1.34) | 1.00 (0.73, 1.37) | 1.09 (0.80, 1.48) | 1.16 (0.86, 1.58) | 0.23 |
| Multivariable HR^2^ (95% CI) | 1.00 (ref) | 1.05 (0.76, 1.45) | 0.94 (0.66, 1.35) | 1.10 (0.75, 1.59) | 1.20 (0.76, 1.89) | 0.61 |
| Multivariable HR^3^ (95% CI) | 1.00 (ref) | 1.02 (0.73, 1.42) | 0.90 (0.62, 1.30) | 1.02 (0.69, 1.52) | 1.07 (0.66, 1.73) | 0.99 |
| **Folate** | **Q1** | **Q2** | **Q3** | **Q4** | **Q5** | **P _trend_** |
| Median Intake | 11.72 | 12.58 | 13.17 | 13.78 | 14.72 |  |
| Number of Cases | 73 | 86 | 91 | 84 | 72 |  |
| Age-adjusted HR^1^ (95% CI) | 1.00 (ref) | 1.18 (0.86, 1.61) | 1.26 (0.92, 1.71) | 1.16 (0.85, 1.59) | 1.00 (0.72, 1.38) | 0.94 |
| Multivariable HR^2^ (95% CI) | 1.00 (ref) | 1.17 (0.86, 1.61) | 1.39 (1.01, 1.90) | 1.34 (0.96, 1.87) | 1.40 (0.99, 1.98) | 0.04 |
| Multivariable HR^3^ (95% CI) | 1.00 (ref) | 1.06 (0.76, 1.48) | 1.19 (0.84, 1.69) | 1.12 (0.76, 1.64) | 1.09 (0.70, 1.69) | 0.65 |
| **Vitamin E** | **Q1** | **Q2** | **Q3** | **Q4** | **Q5** | **P _trend_** |
| Median Intake | 1.85 | 2.09 | 2.26 | 2.43 | 2.71 |  |
| Number of Cases | 90 | 98 | 81 | 70 | 65 |  |
| Age-adjusted HR^1^ (95% CI) | 1.00 (ref) | 1.09 (0.82, 1.45) | 0.91 (0.67, 1.23) | 0.79 (0.58, 1.08) | 0.74 (0.54, 1.02) | 0.01 |
| Multivariable HR^2^ (95% CI) | 1.00 (ref) | 1.10 (0.82, 1.48) | 1.00 (0.73, 1.37) | 0.98 (0.71, 1.36) | 0.95 (0.68, 1.33) | 0.60 |
| Multivariable HR^3^ (95% CI) | 1.00 (ref) | 1.08 (0.80, 1.45) | 0.97 (0.70, 1.34) | 0.93 (0.66, 1.31) | 0.84 (0.59, 1.22) | 0.26 |
| **Vitamin D** | **Q1** | **Q2** | **Q3** | **Q4** | **Q5** | **P _trend_** |
| Median Intake | 0.58 | 1.14 | 1.51 | 1.89 | 2.46 |  |
| Number of Cases | 79 | 79 | 99 | 72 | 76 |  |
| Age-adjusted HR^1^ (95% CI) | 1.00 (ref) | 1.01 (0.74, 1.40) | 1.28 (0.93, 1.78) | 0.92 (0.63, 1.34) | 0.90 (0.57, 1.41) | 0.77 |
| Multivariable HR^2^ (95% CI) | 1.00 (ref) | 1.10 (0.79, 1.52) | 1.34 (0.96, 1.88) | 1.02 (0.69, 1.51) | 1.07 (0.67, 1.70) | 0.68 |
| Multivariable HR^3^ (95% CI) | 1.00 (ref) | 1.10 (0.79, 1.53) | 1.37 (0.97, 1.94) | 1.04 (0.69, 1.55) | 1.11 (0.69, 1.78) | 0.59 |
| **Magnesium** | **Q1** | **Q2** | **Q3** | **Q4** | **Q5** | **P _trend_** |
| Median Intake | 10.14 | 10.72 | 11.11 | 11.49 | 12.03 |  |
| Number of Cases | 104 | 81 | 81 | 67 | 70 |  |
| Age-adjusted HR^1^ (95% CI) | 1.00 (ref) | 0.78 (0.59, 1.05) | 0.79 (0.59, 1.06) | 0.66 (0.48, 0.89) | 0.69 (0.51, 0.93) | 0.01 |
| Multivariable HR^2^ (95% CI) | 1.00 (ref) | 0.85 (0.63, 1.14) | 0.99 (0.73, 1.34) | 0.92 (0.66, 1.27) | 1.11 (0.79, 1.56) | 0.51 |
| Multivariable HR^3^ (95% CI) | 1.00 (ref) | 0.77 (0.57, 1.06) | 0.86 (0.62, 1.19) | 0.76 (0.52, 1.09) | 0.80 (0.53, 1.22) | 0.33 |
| **Zinc** | **Q1** | **Q2** | **Q3** | **Q4** | **Q5** | **P _trend_** |
| Median Intake | 2.24 | 2.54 | 2.75 | 2.95 | 3.24 |  |
| Number of Cases | 108 | 92 | 71 | 65 | 70 |  |
| Age-adjusted HR^1^ (95% CI) | 1.00 (ref) | 0.86 (0.65, 1.13) | 0.67 (0.49, 0.90) | 0.62 (0.45, 0.84) | 0.67 (0.50, 0.90) | <0.01 |
| Multivariable HR^2^ (95% CI) | 1.00 (ref) | 0.86 (0.65, 1.15) | 0.81 (0.60, 1.11) | 0.88 (0.63, 1.24) | 1.05 (0.75, 1.50) | 0.94 |
| Multivariable HR^3^ (95% CI) | 1.00 (ref) | 0.85 (0.63, 1.14) | 0.78 (0.56, 1.09) | 0.80 (0.55, 1.17) | 0.96 (0.64, 1.45) | 0.68 |

^1^ Adjusted for entry age ^2^Adjusted for entry age, sex (overall), calories, smoking status, race, education, BMI, and physical activity ^3^Additionally adjusted for

vitamin C, vitamin E, beta-carotene, and folate

**Table S4 – Hazard Ratios (HRs) and corresponding 95% confidence intervals (CIs) for papillary thyroid cancer by quintile of micronutrient intake among men in The NIH-AARP Diet and Health Study:**

| **Selenium** | **Q1** | **Q2** | **Q3** | **Q4** | **Q5** | **P _trend_** |
| --- | --- | --- | --- | --- | --- | --- |
| Median Intake | 7.05 | 7.64 | 8.03 | 8.41 | 8.93 |  |
| Number of Cases | 12 | 14 | 33 | 49 | 56 |  |
| Age-adjusted HR^1^ (95% CI) | 1.00 (ref) | 0.65 (0.30, 1.41) | 1.01 (0.52, 1.96) | 1.17 (0.62, 2.20) | 1.16 (0.62, 2.17) | 0.14 |
| Multivariable HR^2^ (95% CI) | 1.00 (ref) | 0.62 (0.29, 1.34) | 0.87 (0.44, 1.70) | 1.05 (0.56, 1.98) | 1.01 (0.54, 1.89) | 0.30 |
| Multivariable HR^3^ (95% CI) | 1.00 (ref) | 0.76 (0.34, 1.72) | 1.06 (0.51, 2.20) | 1.36 (0.67, 2.75) | 1.32 (0.65, 2.69) | 0.10 |
| **Vitamin C** | **Q1** | **Q2** | **Q3** | **Q4** | **Q5** | **P _trend_** |
| Median Intake | 7 | 8.41 | 9.36 | 10.27 | 11.67 |  |
| Number of Cases | 20 | 24 | 29 | 52 | 37 |  |
| Age-adjusted HR^1^ (95% CI) | 1.00 (ref) | 1.21 (0.67, 2.19) | 1.46 (0.83, 2.59) | 2.58 (1.54, 4.32) | 1.70 (0.98, 2.93) | <0.01 |
| Multivariable HR^2^ (95% CI) | 1.00 (ref) | 1.17 (0.64, 2.12) | 1.27 (0.70, 2.28) | 2.45 (1.46, 4.15) | 1.70 (0.98, 2.98) | 0.01 |
| Multivariable HR^3^ (95% CI) | 1.00 (ref) | 1.19 (0.65, 2.19) | 1.31 (0.71, 2.44) | 2.55 (1.42, 4.59) | 1.88 (0.97, 3.64) | 0.01 |
| **Betacarotene** | **Q1** | **Q2** | **Q3** | **Q4** | **Q5** | **P _trend_** |
| Median Intake | 8.67 | 9.38 | 9.89 | 10.43 | 11.3 |  |
| Number of Cases | 31 | 31 | 33 | 34 | 33 |  |
| Age-adjusted HR^1^ (95% CI) | 1.00 (ref) | 1.01 (0.61, 1.66) | 1.10 (0.68, 1.80) | 1.18 (0.73, 1.93) | 1.24 (0.76, 2.04) | 0.29 |
| Multivariable HR^2^ (95% CI) | 1.00 (ref) | 0.94 (0.57, 1.55) | 1.02 (0.62, 1.68) | 1.14 (0.70, 1.86) | 1.18 (0.71, 1.95) | 0.39 |
| Multivariable HR^3^ (95% CI) | 1.00 (ref) | 0.87 (0.52, 1.46) | 0.89 (0.53, 1.51) | 0.91 (0.53, 1.57) | 0.93 (0.52, 1.67) | 0.86 |
| **Calcium** | **Q1** | **Q2** | **Q3** | **Q4** | **Q5** | **P _trend_** |
| Median Intake | 8.67 | 9.38 | 9.89 | 10.43 | 11.3 |  |
| Number of Cases | 31 | 31 | 33 | 34 | 33 |  |
| Age-adjusted HR^1^ (95% CI) | 1.00 (ref) | 1.01 (0.61, 1.66) | 1.10 (0.68, 1.80) | 1.18 (0.73, 1.93) | 1.24 (0.76, 2.04) | 0.29 |
| Multivariable HR^2^ (95% CI) | 1.00 (ref) | 1.58 (0.86, 2.90) | 1.57 (0.83, 2.95) | 2.01 (1.05, 3.88) | 1.94 (0.90, 4.17) | 0.12 |
| Multivariable HR^3^ (95% CI) | 1.00 (ref) | 1.48 (0.79, 2.80) | 1.45 (0.75, 2.83) | 1.81 (0.90, 3.63) | 1.60 (0.71, 3.59) | 0.36 |
| **Folate** | **Q1** | **Q2** | **Q3** | **Q4** | **Q5** | **P _trend_** |
| Median Intake | 11.72 | 12.58 | 13.17 | 13.78 | 14.72 |  |
| Number of Cases | 12 | 26 | 34 | 41 | 50 |  |
| Age-adjusted HR^1^ (95% CI) | 1.00 (ref) | 1.62 (0.82, 3.22) | 1.81 (0.94, 3.49) | 1.78 (0.94, 3.40) | 1.90 (1.01, 3.57) | 0.08 |
| Multivariable HR^2^ (95% CI) | 1.00 (ref) | 1.60 (0.81, 3.19) | 1.74 (0.90, 3.38) | 1.69 (0.88, 3.26) | 1.97 (1.04, 3.75) | 0.08 |
| Multivariable HR^3^ (95% CI) | 1.00 (ref) | 1.36 (0.68, 2.75) | 1.29 (0.64, 2.62) | 1.18 (0.57, 2.45) | 1.21 (0.55, 2.62) | 0.89 |
| **Vitamin E** | **Q1** | **Q2** | **Q3** | **Q4** | **Q5** | **P _trend_** |
| Median Intake | 1.85 | 2.09 | 2.26 | 2.43 | 2.71 |  |
| Number of Cases | 22 | 29 | 29 | 43 | 41 |  |
| Age-adjusted HR^1^ (95% CI) | 1.00 (ref) | 1.04 (0.60, 1.82) | 0.83 (0.48, 1.45) | 1.06 (0.64, 1.78) | 0.94 (0.56, 1.57) | 0.86 |
| Multivariable HR^2^ (95% CI) | 1.00 (ref) | 1.03 (0.59, 1.80) | 0.79 (0.45, 1.40) | 1.03 (0.61, 1.74) | 0.93 (0.55, 1.59) | 0.02 |
| Multivariable HR^3^ (95% CI) | 1.00 (ref) | 0.96 (0.54, 1.72) | 0.72 (0.40, 1.30) | 0.90 (0.51, 1.58) | 0.78 (0.44, 1.40) | 0.44 |
| **Vitamin D** | **Q1** | **Q2** | **Q3** | **Q4** | **Q5** | **P _trend_** |
| Median Intake | 0.58 | 1.14 | 1.51 | 1.89 | 2.46 |  |
| Number of Cases | 22 | 32 | 38 | 35 | 37 |  |
| Age-adjusted HR^1^ (95% CI) | 1.00 (ref) | 1.05 (0.60, 1.82) | 1.00 (0.57, 1.75) | 0.78 (0.42, 1.42) | 0.73 (0.36, 1.48) | 0.22 |
| Multivariable HR^2^ (95% CI) | 1.00 (ref) | 1.05 (0.60, 1.83) | 0.97 (0.55, 1.71) | 0.73 (0.40, 1.35) | 0.70 (0.34, 1.43) | 0.17 |
| Multivariable HR^3^ (95% CI) | 1.00 (ref) | 1.10 (0.71, 1.97) | 1.06 (0.58, 1.91) | 0.81 (0.43, 1.53) | 0.79 (0.38, 1.67) | 0.31 |
| **Magnesium** | **Q1** | **Q2** | **Q3** | **Q4** | **Q5** | **P _trend_** |
| Median Intake | 10.14 | 10.72 | 11.11 | 11.49 | 12.03 |  |
| Number of Cases | 15 | 23 | 32 | 39 | 54 |  |
| Age-adjusted HR^1^ (95% CI) | 1.00 (ref) | 1.01 (0.53, 1.94) | 1.09 (0.59, 2.02) | 1.10 (0.61, 1.99) | 1.30 (0.73, 2.3) | 0.25 |
| Multivariable HR^2^ (95% CI) | 1.00 (ref) | 1.01 (0.52, 1.97) | 1.13 (0.60, 2.12) | 1.10 (0.59, 2.03) | 1.35 (0.75 2.45) | 0.20 |
| Multivariable HR^3^ (95% CI) | 1.00 (ref) | 1.00 (0.50, 2.02) | 1.06 (0.54, 2.08) | 0.97 (0.49, 1.94) | 1.06 (0.52, 2.17) | 0.83 |
| **Zinc** | **Q1** | **Q2** | **Q3** | **Q4** | **Q5** | **P _trend_** |
| Median Intake | 2.24 | 2.54 | 2.75 | 2.95 | 3.24 |  |
| Number of Cases | 15 | 14 | 29 | 45 | 61 |  |
| Age-adjusted HR^1^ (95% CI) | 1.00 (ref) | 0.55 (0.27, 1.15) | 0.74 (0.40, 1.39) | 0.87 (0.49, 1.57) | 1.06 (0.60, 1.87) | 0.12 |
| Multivariable HR^2^ (95% CI) | 1.00 (ref) | 0.47 (0.22, 0.99) | 0.67 (0.36, 1.25) | 0.75 (0.42, 1.36) | 0.93 (0.52, 1.64) | 0.23 |
| Multivariable HR^3^ (95% CI) | 1.00 (ref) | 0.49 (0.22, 1.08) | 0.67 (0.33, 1.35) | 0.74 (0.37, 1.48) | 0.87 (0.42, 1.80) | 0.36 |

^1^ Adjusted for entry age ^2^Adjusted for entry age, sex (overall), calories, smoking status, race, education, BMI, and physical activity ^3^Additionally adjusted for

vitamin C, vitamin E, beta-carotene, and folate

**Table S5 – Hazard Ratios (HRs) and corresponding 95% confidence intervals (CIs) for papillary thyroid cancer by quintile of micronutrient intake among women in The NIH-AARP Diet and Health Study:**

| **Selenium** | **Q1** | **Q2** | **Q3** | **Q4** | **Q5** | **P _trend_** |
| --- | --- | --- | --- | --- | --- | --- |
| Median Intake | 7.05 | 7.64 | 8.03 | 8.41 | 8.93 |  |
| Number of Cases | 83 | 77 | 49 | 21 | 12 |  |
| Age-adjusted HR^1^ (95% CI) | 1.00 (ref) | 1.20 (0.88, 1.64) | 1.19 (0.84, 1.70) | 0.96 (0.60, 1.55) | 1.29 (0.71, 2.37) | 0.45 |
| Multivariable HR^2^ (95% CI) | 1.00 (ref) | 1.14 (0.83, 1.57) | 1.09 (0.76, 1.58) | 0.92 (0.56, 1.50) | 1.29 (0.70, 2.38) | 0.65 |
| Multivariable HR^3^ (95% CI) | 1.00 (ref) | 1.21 (0.87, 1.67) | 1.17 (0.80, 1.72) | 0.99 (0.60, 1.65) | 1.29 (0.68, 2.46) | 0.48 |
| **Vitamin C** | **Q1** | **Q2** | **Q3** | **Q4** | **Q5** | **P _trend_** |
| Median Intake | 7 | 8.41 | 9.36 | 10.27 | 11.67 |  |
| Number of Cases | 20 | 24 | 29 | 52 | 37 |  |
| Age-adjusted HR^1^ (95% CI) | 1.00 (ref) | 1.21 (0.67, 2.19) | 1.46 (0.83, 2.59) | 2.58 (1.54, 4.32) | 1.70 (0.98, 2.93) | <0.01 |
| Multivariable HR^2^ (95% CI) | 1.00 (ref) | 1.17 (0.64, 2.12) | 1.27 (0.70, 2.28) | 2.45 (1.46, 4.15) | 1.70 (0.98, 2.98) | 0.01 |
| Multivariable HR^3^ (95% CI) | 1.00 (ref) | 1.19 (0.65, 2.19) | 1.31 (0.71, 2.44) | 2.55 (1.42, 4.59) | 1.88 (0.97, 3.64) | 0.01 |
| **Betacarotene** | **Q1** | **Q2** | **Q3** | **Q4** | **Q5** | **P _trend_** |
| Median Intake | 8.67 | 9.38 | 9.89 | 10.43 | 11.3 |  |
| Number of Cases | 46 | 44 | 44 | 50 | 57 |  |
| Age-adjusted HR^1^ (95% CI) | 1.00 (ref) | 0.94 (0.62, 1.42) | 0.89 (0.59, 1.35) | 0.95 (0.64, 1.42) | 0.98 (0.66, 1.44) | 0.99 |
| Multivariable HR^2^ (95% CI) | 1.00 (ref) | 0.94 (0.62, 1.43) | 0.81 (0.53, 1.25) | 0.94 (0.62, 1.42) | 0.96 (0.64, 1.45) | 0.88 |
| Multivariable HR^3^ (95% CI) | 1.00 (ref) | 0.85 (0.56, 1.31) | 0.72 (0.46, 1.12) | 0.79 (0.51, 1.24) | 0.76 (0.47, 1.22) | 0.25 |
| **Calcium** | **Q1** | **Q2** | **Q3** | **Q4** | **Q5** | **P _trend_** |
| Median Intake | 8.67 | 9.38 | 9.89 | 10.43 | 11.3 |  |
| Number of Cases | 46 | 44 | 44 | 50 | 57 |  |
| Age-adjusted HR^1^ (95% CI) | 1.00 (ref) | 0.94 (0.62, 1.42) | 0.89 (0.59, 1.35) | 0.95 (0.64, 1.42) | 0.98 (0.66, 1.44) | 0.99 |
| Multivariable HR^2^ (95% CI) | 1.00 (ref) | 0.88 (0.59, 1.30) | 0.73 (0.47, 1.15) | 0.78 (0.48, 1.26) | 0.97 (0.54, 1.73) | 0.53 |
| Multivariable HR^3^ (95% CI) | 1.00 (ref) | 0.86 (0.58, 1.29) | 0.71 (0.44, 1.12) | 0.74 (0.45, 1.23) | 0.89 (0.48, 1.66) | 0.42 |
| **Folate** | **Q1** | **Q2** | **Q3** | **Q4** | **Q5** | **P _trend_** |
| Median Intake | 11.72 | 12.58 | 13.17 | 13.78 | 14.72 |  |
| Number of Cases | 61 | 60 | 56 | 43 | 22 |  |
| Age-adjusted HR^1^ (95% CI) | 1.00 (ref) | 1.22 (0.85, 1.74) | 1.43 (0.99, 2.05) | 1.49 (1.01, 2.20) | 1.14 (0.70, 1.86) | 0.13 |
| Multivariable HR^2^ (95% CI) | 1.00 (ref) | 1.07 (0.74, 1.54) | 1.33 (0.92, 1.92) | 1.31 (0.87, 1.96) | 1.15 (0.70, 1.88) | 0.19 |
| Multivariable HR^3^ (95% CI) | 1.00 (ref) | 1.01 (0.68, 1.49) | 1.24 (0.81,1.88) | 1.20 (0.74, 1.95) | 1.05 (0.57, 1.93) | 0.45 |
| **Vitamin E** | **Q1** | **Q2** | **Q3** | **Q4** | **Q5** | **P _trend_** |
| Median Intake | 1.85 | 2.09 | 2.26 | 2.43 | 2.71 |  |
| Number of Cases | 68 | 69 | 52 | 27 | 24 |  |
| Age-adjusted HR^1^ (95% CI) | 1.00 (ref) | 1.21 (0.87, 1.70) | 1.21 (0.84, 1.74) | 0.85 (0.54, 1.33) | 0.96 (0.60, 1.53) | 0.67 |
| Multivariable HR^2^ (95% CI) | 1.00 (ref) | 1.13 (0.80, 1.60) | 1.16 (0.80, 1.68) | 0.88 (0.56, 1.37) | 0.91 (0.56, 1.48) | 0.61 |
| Multivariable HR^3^ (95% CI) | 1.00 (ref) | 1.13 (0.80, 1.60) | 1.15 (0.79, 1.69) | 0.86 (0.54, 1.37) | 0.80 (0.48, 1.35) | 0.38 |
| **Vitamin D** | **Q1** | **Q2** | **Q3** | **Q4** | **Q5** | **P _trend_** |
| Median Intake | 0.58 | 1.14 | 1.51 | 1.89 | 2.46 |  |
| Number of Cases | 57 | 47 | 61 | 37 | 39 |  |
| Age-adjusted HR^1^ (95% CI) | 1.00 (ref) | 1.07 (0.72, 1.60) | 1.72 (1.15, 2.57) | 1.25 (0.76, 2.04) | 1.40 (0.77, 2.54) | 0.10 |
| Multivariable HR^2^ (95% CI) | 1.00 (ref) | 1.11 (0.74, 1.67) | 1.63 (1.07, 2.48) | 1.24 (0.75, 2.07) | 1.44 (0.78, 2.65) | 0.10 |
| Multivariable HR^3^ (95% CI) | 1.00 (ref) | 1.09 (0.72, 1.64) | 1.63 (1.06, 2.50) | 1.21 (0.72, 2.05) | 1.43 (0.76, 2.69) | 0.13 |
| **Magnesium** | **Q1** | **Q2** | **Q3** | **Q4** | **Q5** | **P _trend_** |
| Median Intake | 10.14 | 10.72 | 11.11 | 11.49 | 12.03 |  |
| Number of Cases | 89 | 58 | 49 | 28 | 16 |  |
| Age-adjusted HR^1^ (95% CI) | 1.00 (ref) | 0.84 (0.61, 1.18) | 0.96 (0.68, 1.36) | 0.81 (0.53, 1.24) | 0.87 (0.51, 1.48) | 0.41 |
| Multivariable HR^2^ (95% CI) | 1.00 (ref) | 0.82 (0.58, 1.16) | 1.00 (0.70, 1.43) | 0.88 (0.57, 1.36) | 0.98 (0.57, 1.68) | 0.85 |
| Multivariable HR^3^ (95% CI) | 1.00 (ref) | 0.74 (0.52, 1.06) | 0.84 (0.57, 1.25) | 0.71 (0.44, 1.15) | 0.66 (0.35, 1.26) | 0.15 |
| **Zinc** | **Q1** | **Q2** | **Q3** | **Q4** | **Q5** | **P _trend_** |
| Median Intake | 2.24 | 2.54 | 2.75 | 2.95 | 3.24 |  |
| Number of Cases | 93 | 78 | 42 | 20 | 9 |  |
| Age-adjusted HR^1^ (95% CI) | 1.00 (ref) | 1.06 (0.78, 1.43) | 0.89 (0.62, 1.28) | 0.87 (0.54, 1.41) | 0.82 (0.42, 1.63) | 0.4 |
| Multivariable HR^2^ (95% CI) | 1.00 (ref) | 0.98 (0.72, 1.33) | 0.84 (0.58, 1.22) | 0.85 (0.52, 1.38) | 0.81 (0.41, 1.61) | 0.35 |
| Multivariable HR^3^ (95% CI) | 1.00 (ref) | 0.95 (0.69, 1.30) | 0.79 (0.53, 1.17) | 0.70 (0.41, 1.20) | 0.75 (0.36, 1.57) | 0.15 |

^1^ Adjusted for entry age ^2^Adjusted for entry age, sex (overall), calories, smoking status, race, education, BMI, and physical activity ^3^Additionally adjusted for

vitamin C, vitamin E, beta-carotene, and folate

**Table S6 – Hazard Ratios (HRs) and corresponding 95% confidence intervals (CIs) for follicular thyroid cancer by quintile of micronutrient intake among men and women combined in The NIH-AARP Diet and Health Study:**

| **Selenium** | **Q1** | **Q2** | **Q3** | **Q4** | **Q5** | **P _trend_** |
| --- | --- | --- | --- | --- | --- | --- |
| Median Intake | 7.05 | 7.64 | 8.03 | 8.41 | 8.93 |  |
| Number of Cases | 25 | 22 | 22 | 19 | 25 |  |
| Age-adjusted HR^1^ (95% CI) | 1.00 (ref) | 0.89 (0.50, 1.58) | 0.90 (0.51, 1.59) | 0.79 (0.44, 1.43) | 1.05 (0.60, 1.83) | 0.67 |
| Multivariable HR^2^ (95% CI) | 1.00 (ref) | 0.96 (0.54, 1.73) | 1.05 (0.58, 1.93) | 1.00 (0.52, 1.92) | 1.41 (0.73, 2.69) | 0.35 |
| Multivariable HR^3^ (95% CI) | 1.00 (ref) | 0.98 (0.54, 1.78) | 1.07 (0.57, 1.98) | 1.00 (0.51, 1.98) | 1.41 (0.71, 2.79) | 0.37 |
| **Vitamin C** | **Q1** | **Q2** | **Q3** | **Q4** | **Q5** | **P _trend_** |
| Median Intake | 7 | 8.41 | 9.36 | 10.27 | 11.67 |  |
| Number of Cases | 8 | 13 | 11 | 10 | 15 |  |
| Age-adjusted HR^1^ (95% CI) | 1.00 (ref) | 1.59 (0.66, 3.83) | 1.31 (0.53, 3.27) | 1.16 (0.46, 2.93) | 1.61 (0.68, 3.81) | 0.46 |
| Multivariable HR^2^ (95% CI) | 1.00 (ref) | 1.02 (0.57, 1.80) | 0.68 (0.36, 1.27) | 0.75 (0.40, 1.39) | 1.11 (0.63, 1.97) | <0.01 |
| Multivariable HR^3^ (95% CI) | 1.00 (ref) | 0.97 (0.54, 1.75) | 0.64 (0.33, 1.26) | 0.71 (0.36, 1.42) | 1.09 (0.53, 2.22) | 0.96 |
| **Betacarotene** | **Q1** | **Q2** | **Q3** | **Q4** | **Q5** | **P _trend_** |
| Median Intake | 8.67 | 9.38 | 9.89 | 10.43 | 11.3 |  |
| Number of Cases | 14 | 29 | 22 | 22 | 26 |  |
| Age-adjusted HR^1^ (95% CI) | 1.00 (ref) | 2.01 (1.06, 3.81) | 1.50 (0.77, 2.94) | 1.48 (0.76, 2.90) | 1.74 (0.91, 3.35) | 0.32 |
| Multivariable HR^2^ (95% CI) | 1.00 (ref) | 1.98 (1.04, 3.75) | 1.47 (0.74, 2.86) | 1.45 (0.74, 2.86) | 1.72 (0.89, 3.33) | 0.39 |
| Multivariable HR^3^ (95% CI) | 1.00 (ref) | 2.02 (1.06, 3.86) | 1.46 (0.72, 2.95) | 1.54 (0.75, 3.17) | 1.92 (0.91, 4.10) | 0.31 |
| **Calcium** | **Q1** | **Q2** | **Q3** | **Q4** | **Q5** | **P _trend_** |
| Median Intake | 8.67 | 9.38 | 9.89 | 10.43 | 11.3 |  |
| Number of Cases | 14 | 29 | 22 | 22 | 26 |  |
| Age-adjusted HR^1^ (95% CI) | 1.00 (ref) | 2.01 (1.06, 3.81) | 1.50 (0.77, 2.94) | 1.48 (0.76, 2.90) | 1.74 (0.91, 3.35) | 0.32 |
| Multivariable HR^2^ (95% CI) | 1.00 (ref) | 0.79 (0.40, 1.56) | 1.04 (0.53, 2.06) | 1.66 (0.84, 3.28) | 1.18 (0.50, 2.80) | 0.37 |
| Multivariable HR^3^ (95% CI) | 1.00 (ref) | 0.78 (0.39, 1.55) | 1.04 (0.52, 2.09) | 1.68 (0.83, 3.40) | 1.16 (0.47, 2.86) | 0.43 |
| **Folate** | **Q1** | **Q2** | **Q3** | **Q4** | **Q5** | **P _trend_** |
| Median Intake | 11.72 | 12.58 | 13.17 | 13.78 | 14.72 |  |
| Number of Cases | 30 | 18 | 21 | 15 | 29 |  |
| Age-adjusted HR^1^ (95% CI) | 1.00 (ref) | 0.58 (0.33, 1.05) | 0.68 (0.39, 1.19) | 0.48 (0.26, 0.90) | 0.93 (0.56, 1.55) | 0.70 |
| Multivariable HR^2^ (95% CI) | 1.00 (ref) | 0.60 (0.33, 1.08) | 0.72 (0.41, 1.28) | 0.54 (0.28, 1.03) | 1.12 (0.64, 1.96) | 0.69 |
| Multivariable HR^3^ (95% CI) | 1.00 (ref) | 0.54 (0.29, 1.00) | 0.63 (0.34, 1.19) | 0.46 (0.22, 0.96) | 0.92 (0.44, 1.96) | 0.97 |
| **Vitamin E** | **Q1** | **Q2** | **Q3** | **Q4** | **Q5** | **P _trend_** |
| Median Intake | 1.85 | 2.09 | 2.26 | 2.43 | 2.71 |  |
| Number of Cases | 24 | 24 | 29 | 20 | 16 |  |
| Age-adjusted HR^1^ (95% CI) | 1.00 (ref) | 0.98 (0.56, 1.73) | 1.18 (0.69, 2.04) | 0.81 (0.45, 2.04) | 0.64 (0.34, 1.21) | 0.14 |
| Multivariable HR^2^ (95% CI) | 1.00 (ref) | 1.03 (0.58, 1.82) | 1.28 (0.74, 2.24) | 0.91 (0.49, 1.68) | 0.74 (0.38, 1.43) | 0.35 |
| Multivariable HR^3^ (95% CI) | 1.00 (ref) | 1.01 (0.56, 1.81) | 1.23 (0.69, 2.19) | 0.85 (0.45, 1.62) | 0.69 (0.34, 1.40) | 0.26 |
| **Vitamin D** | **Q1** | **Q2** | **Q3** | **Q4** | **Q5** | **P _trend_** |
| Median Intake | 0.58 | 1.14 | 1.51 | 1.89 | 2.46 |  |
| Number of Cases | 20 | 19 | 24 | 32 | 18 |  |
| Age-adjusted HR^1^ (95% CI) | 1.00 (ref) | 0.94 (0.49, 1.79) | 1.05 (0.54, 2.03) | 1.18 (0.60, 2.34) | 0.68 (0.29, 1.61) | 0.65 |
| Multivariable HR^2^ (95% CI) | 1.00 (ref) | 0.94 (0.49, 1.80) | 1.06 (0.55, 2.06) | 1.23 (0.62, 2.44) | 0.70 (0.29, 1.68) | 0.71 |
| Multivariable HR^3^ (95% CI) | 1.00 (ref) | 0.91 (0.48, 1.75) | 1.03 (0.53, 2.02) | 1.19 (0.59, 2.40) | 0.64 (0.26, 1.59) | 0.63 |
| **Magnesium** | **Q1** | **Q2** | **Q3** | **Q4** | **Q5** | **P _trend_** |
| Median Intake | 10.14 | 10.72 | 11.11 | 11.49 | 12.03 |  |
| Number of Cases | 23 | 21 | 22 | 27 | 20 |  |
| Age-adjusted HR^1^ (95% CI) | 1.00 (ref) | 0.90 (0.50, 1.62) | 0.94 (0.52, 1.68) | 1.16 (0.66, 2.02) | 0.86 (0.47, 1.56) | 0.91 |
| Multivariable HR^2^ (95% CI) | 1.00 (ref) | 0.94 (0.52, 1.70) | 1.03 (0.56, 1.87) | 1.33 (0.74, 2.40) | 1.05 (0.55, 2.02) | 0.63 |
| Multivariable HR^3^ (95% CI) | 1.00 (ref) | 0.92 (0.50, 1.69) | 0.99 (0.52, 1.87) | 1.22 (0.63, 2.38) | 1.00 (0.46, 2.18) | 0.88 |
| **Zinc** | **Q1** | **Q2** | **Q3** | **Q4** | **Q5** | **P _trend_** |
| Median Intake | 2.24 | 2.54 | 2.75 | 2.95 | 3.24 |  |
| Number of Cases | 23 | 30 | 17 | 25 | 18 |  |
| Age-adjusted HR^1^ (95% CI) | 1.00 (ref) | 1.30 (0.76, 2.24) | 0.75 (0.40, 1.40) | 1.11 (0.63, 1.95) | 0.81 (0.44, 1.50) | 0.40 |
| Multivariable HR^2^ (95% CI) | 1.00 (ref) | 1.32 (0.76, 2.29) | 0.80 (0.42, 1.54) | 1.26 (0.68, 2.36) | 0.95 (0.47, 1.90) | 0.81 |
| Multivariable HR^3^ (95% CI) | 1.00 (ref) | 1.29 (0.73, 2.29) | 0.77 (0.39, 1.52) | 1.18 (0.59, 2.37) | 0.91 (0.41, 2.03) | 0.63 |

^1^ Adjusted for entry age ^2^Adjusted for entry age, sex (overall), calories, smoking status, race, education, BMI, and physical activity ^3^Additionally adjusted for

vitamin C, vitamin E, beta-carotene, and folate

**Table S7 – Hazard Ratios (HRs) and corresponding 95% confidence intervals (CIs) for follicular thyroid cancer by quintile of micronutrient intake among men in The NIH-AARP Diet and Health Study:**

| **Selenium** | **Q1** | **Q2** | **Q3** | **Q4** | **Q5** | **P _trend_** |
| --- | --- | --- | --- | --- | --- | --- |
| Median Intake | 7.05 | 7.64 | 8.03 | 8.41 | 8.93 |  |
| Number of Cases | 4 | 8 | 8 | 14 | 23 |  |
| Age-adjusted HR^1^ (95% CI) | 1.00 (ref) | 1.11 (0.33, 3.70) | 0.73 (0.22, 2.44) | 1.02 (0.34, 3.09) | 1.48 (0.51, 4.29) | 0.23 |
| Multivariable HR^2^ (95% CI) | 1.00 (ref) | 1.03 (0.31, 3.43) | 0.66 (0.20, 2.20) | 0.91 (0.30, 2.78) | 1.35 (0.46, 3.92) | 0.28 |
| Multivariable HR^3^ (95% CI) | 1.00 (ref) | 1.03 (0.31, 3.46) | 0.66 (0.19, 2.23) | 0.90 (0.29, 2.83) | 1.32 (0.43, 4.03) | 0.33 |
| **Vitamin C** | **Q1** | **Q2** | **Q3** | **Q4** | **Q5** | **P _trend_** |
| Median Intake | 7 | 8.41 | 9.36 | 10.27 | 11.67 |  |
| Number of Cases | 8 | 13 | 11 | 10 | 15 |  |
| Age-adjusted HR^1^ (95% CI) | 1.00 (ref) | 1.59 (0.66, 3.83) | 1.31 (0.53, 3.27) | 1.16 (0.46, 2.93) | 1.61 (0.68, 3.81) | 0.46 |
| Multivariable HR^2^ (95% CI) | 1.00 (ref) | 1.50 (0.62, 3.64) | 1.24 (0.49, 3.10) | 1.08 (0.42, 2.77) | 1.55 (0.64, 3.71) | 0.40 |
| Multivariable HR^3^ (95% CI) | 1.00 (ref) | 1.41 (0.57, 3.49) | 1.13 (0.43, 2.99) | 0.97 (0.34, 2.72) | 1.40 (0.49, 4.02) | 0.75 |
| **Betacarotene** | **Q1** | **Q2** | **Q3** | **Q4** | **Q5** | **P _trend_** |
| Median Intake | 8.67 | 9.38 | 9.89 | 10.43 | 11.3 |  |
| Number of Cases | 7 | 14 | 13 | 10 | 13 |  |
| Age-adjusted HR^1^ (95% CI) | 1.00 (ref) | 1.96 (0.79, 4.85) | 1.85 (0.74, 4.63) | 1.46 (0.56, 3.85) | 2.05 (0.81, 5.16) | 0.27 |
| Multivariable HR^2^ (95% CI) | 1.00 (ref) | 1.88 (0.76, 4.66) | 1.76 (0.70, 4.43) | 1.41 (0.53, 3.73) | 2.01 (0.79, 5.10) | 0.30 |
| Multivariable HR^3^ (95% CI) | 1.00 (ref) | 1.84 (0.73, 4.64) | 1.71 (0.66, 4.47) | 1.37 (0.48, 3.85) | 1.97 (0.69, 5.65) | 0.40 |
| **Calcium** | **Q1** | **Q2** | **Q3** | **Q4** | **Q5** | **P _trend_** |
| Median Intake | 8.67 | 9.38 | 9.89 | 10.43 | 11.3 |  |
| Number of Cases | 7 | 14 | 13 | 10 | 13 |  |
| Age-adjusted HR^1^ (95% CI) | 1.00 (ref) | 1.96 (0.79, 4.85) | 1.85 (0.74, 4.63) | 1.46 (0.56, 3.85) | 2.05 (0.81, 5.16) | 0.27 |
| Multivariable HR^2^ (95% CI) | 1.00 (ref) | 0.68 (0.26, 1.83) | 0.62 (0.23, 1.68) | 0.79 (0.29, 2.16) | 0.84 (0.26, 2.70) | 0.74 |
| Multivariable HR^3^ (95% CI) | 1.00 (ref) | 0.64 (0.24, 1.72) | 0.56 (0.20, 1.56) | 0.70 (0.24, 1.99) | 0.72 (0.21, 2.44) | 0.58 |
| **Folate** | **Q1** | **Q2** | **Q3** | **Q4** | **Q5** | **P _trend_** |
| Median Intake | 11.72 | 12.58 | 13.17 | 13.78 | 14.72 |  |
| Number of Cases | 8 | 7 | 10 | 11 | 21 |  |
| Age-adjusted HR^1^ (95% CI) | 1.00 (ref) | 0.64 (0.23, 1.76) | 0.74 (0.29, 1.88) | 0.68 (0.27, 1.69) | 1.12 (0.50, 2.54) | 0.43 |
| Multivariable HR^2^ (95% CI) | 1.00 (ref) | 0.60 (0.22, 1.66) | 0.70 (0.27, 1.78) | 0.64 (0.25, 1.61) | 1.10 (0.47, 2.54) | 0.43 |
| Multivariable HR^3^ (95% CI) | 1.00 (ref) | 0.54 (0.19, 1.53) | 0.58 (0.21, 1.61) | 0.51 (0.17, 1.46) | 0.85 (0.28, 2.53) | 0.79 |
| **Vitamin E** | **Q1** | **Q2** | **Q3** | **Q4** | **Q5** | **P _trend_** |
| Median Intake | 1.85 | 2.09 | 2.26 | 2.43 | 2.71 |  |
| Number of Cases | 6 | 12 | 14 | 12 | 13 |  |
| Age-adjusted HR^1^ (95% CI) | 1.00 (ref) | 1.56 (0.59, 4.16) | 1.44 (0.55, 3.75) | 1.05 (0.39, 2.79) | 1.03 (0.39, 2.70) | 0.56 |
| Multivariable HR^2^ (95% CI) | 1.00 (ref) | 1.46 (0.55, 3.90) | 1.36 (0.52, 3.54) | 1.00 (0.37, 2.68) | 1.01 (0.38, 2.67) | 0.58 |
| Multivariable HR^3^ (95% CI) | 1.00 (ref) | 1.28 (0.47, 3.45) | 1.11 (0.42, 2.99) | 0.79 (0.28, 2.20) | 0.77 (0.28, 2.17) | 0.58 |
| **Vitamin D** | **Q1** | **Q2** | **Q3** | **Q4** | **Q5** | **P _trend_** |
| Median Intake | 0.58 | 1.14 | 1.51 | 1.89 | 2.46 |  |
| Number of Cases | 5 | 8 | 13 | 20 | 11 |  |
| Age-adjusted HR^1^ (95% CI) | 1.00 (ref) | 1.39 (0.45, 4.31) | 2.09 (0.69, 6.33) | 2.80 (0.90, 8.69) | 1.31 (0.34, 4.98) | 0.43 |
| Multivariable HR^2^ (95% CI) | 1.00 (ref) | 1.32 (0.42, 4.09) | 1.98 (0.66, 5.96) | 2.64 (0.85, 8.15) | 1.26 (0.33 4.80) | 0.46 |
| Multivariable HR^3^ (95% CI) | 1.00 (ref) | 1.30 (0.42, 4.05) | 1.95 (0.64, 5.93) | 2.62 (0.84, 8.22) | 1.28 (0.33, 4.95) | 0.44 |
| **Magnesium** | **Q1** | **Q2** | **Q3** | **Q4** | **Q5** | **P _trend_** |
| Median Intake | 10.14 | 10.72 | 11.11 | 11.49 | 12.03 |  |
| Number of Cases | 3 | 8 | 13 | 17 | 16 |  |
| Age-adjusted HR^1^ (95% CI) | 1.00 (ref) | 1.73 (0.46, 6.54) | 2.16 (0.62, 7.59) | 2.32 (0.68, 7.93) | 1.86 (0.54, 6.40) | 0.42 |
| Multivariable HR^2^ (95% CI) | 1.00 (ref) | 1.65 (0.44, 6.24) | 2.03 (0.58, 7.17) | 2.18 (0.64, 7.50) | 1.79 (0.52, 6.21) | 0.46 |
| Multivariable HR^3^ (95% CI) | 1.00 (ref) | 1.59 (0.42, 6.08) | 1.87 (0.51, 6.86) | 1.92 (0.52, 7.11) | 1.49 (0.37, 5.90) | 0.83 |
| **Zinc** | **Q1** | **Q2** | **Q3** | **Q4** | **Q5** | **P _trend_** |
| Median Intake | 2.24 | 2.54 | 2.75 | 2.95 | 3.24 |  |
| Number of Cases | 2 | 12 | 8 | 19 | 16 |  |
| Age-adjusted HR^1^ (95% CI) | 1.00 (ref) | 3.51 (0.79, 15.72) | 1.53 (0.33, 7.21) | 2.76 (0.64, 11.86) | 2.10 (0.48, 9.14) | 0.84 |
| Multivariable HR^2^ (95% CI) | 1.00 (ref) | 3.17 (0.70, 14.09) | 1.35 (0.29, 6.38) | 2.42 (0.56, 10.48) | 1.87 (0.43, 8.21) | 0.94 |
| Multivariable HR^3^ (95% CI) | 1.00 (ref) | 2.76 (0.60, 12.67) | 1.10 (0.22, 5.44) | 1.83 (0.39, 8.62) | 1.37 (0.28, 6.82) | 0.61 |

^1^ Adjusted for entry age ^2^Adjusted for entry age, sex (overall), calories, smoking status, race, education, BMI, and physical activity ^3^Additionally adjusted for

vitamin C, vitamin E, beta-carotene, and folate

**Table S8 – Hazard Ratios (HRs) and corresponding 95% confidence intervals (CIs) for follicular thyroid cancer by quintile of micronutrient intake among women in The NIH-AARP Diet and Health Study:**

| **Selenium** | **Q1** | **Q2** | **Q3** | **Q4** | **Q5** | **P _trend_** |
| --- | --- | --- | --- | --- | --- | --- |
| Median Intake | 7.05 | 7.64 | 8.03 | 8.41 | 8.93 |  |
| Number of Cases | 21 | 14 | 14 | 5 | 2 |  |
| Age-adjusted HR^1^ (95% CI) | 1.00 (ref) | 0.87 (0.45, 1.72) | 1.38 (0.70, 2.71) | 0.93 (0.35, 2.48) | 0.88 (0.21, 3.76) | 0.82 |
| Multivariable HR^2^ (95% CI) | 1.00 (ref) | 0.91 (0.46, 1.80) | 1.41 (0.71, 2.81) | 0.94 (0.35, 2.51) | 0.86 (0.20, 3.70) | 0.81 |
| Multivariable HR^3^ (95% CI) | 1.00 (ref) | 0.93 (0.46, 1.87) | 1.43 (0.70, 2.93) | 0.95 (0.35, 2.62) | 0.88 (0.20, 3.87) | 0.80 |
| **Vitamin C** | **Q1** | **Q2** | **Q3** | **Q4** | **Q5** | **P _trend_** |
| Median Intake | 7 | 8.41 | 9.36 | 10.27 | 11.67 |  |
| Number of Cases | 15 | 12 | 7 | 9 | 12 |  |
| Age-adjusted HR^1^ (95% CI) | 1.00 (ref) | 0.76 (0.36, 1.63) | 0.44 (0.18, 1.08) | 0.58 (0.25, 1.32) | 0.87 (0.41, 1.87) | 0.75 |
| Multivariable HR^2^ (95% CI) | 1.00 (ref) | 0.76 (0.35, 1.64) | 0.38 (0.14, 0.98) | 0.58 (0.25, 1.35) | 0.89 (0.41, 1.94) | 0.55 |
| Multivariable HR^3^ (95% CI) | 1.00 (ref) | 0.74 (0.34, 1.64) | 0.37 (0.14, 1.01) | 0.59 (0.23, 1.52) | 0.96 (0.35, 2.64) | 0.69 |
| **Betacarotene** | **Q1** | **Q2** | **Q3** | **Q4** | **Q5** | **P _trend_** |
| Median Intake | 8.67 | 9.38 | 9.89 | 10.43 | 11.3 |  |
| Number of Cases | 7 | 15 | 9 | 12 | 13 |  |
| Age-adjusted HR^1^ (95% CI) | 1.00 (ref) | 2.05 (0.84, 5.03) | 1.15 (0.43, 3.09) | 1.43 (0.56, 3.64) | 1.40 (0.56, 3.51) | 0.87 |
| Multivariable HR^2^ (95% CI) | 1.00 (ref) | 2.12 (0.86, 5.21) | 1.20 (0.45, 3.26) | 1.51 (0.59, 3.88) | 1.52 (0.59, 3.88) | 0.81 |
| Multivariable HR^3^ (95% CI) | 1.00 (ref) | 2.23 (0.90, 5.55) | 1.19 (0.42, 3.38) | 1.77 (0.65, 4.80) | 1.99 (0.69, 5.74) | 0.48 |
| **Calcium** | **Q1** | **Q2** | **Q3** | **Q4** | **Q5** | **P _trend_** |
| Median Intake | 8.67 | 9.38 | 9.89 | 10.43 | 11.3 |  |
| Number of Cases | 7 | 15 | 9 | 12 | 13 |  |
| Age-adjusted HR^1^ (95% CI) | 1.00 (ref) | 2.05 (0.84, 5.03) | 1.15 (0.43, 3.09) | 1.43 (0.56, 3.64) | 1.40 (0.56, 3.51) | 0.87 |
| Multivariable HR^2^ (95% CI) | 1.00 (ref) | 0.81 (0.31, 2.13) | 1.61 (0.65, 3.96) | 3.25 (1.33, 7.94) | 1.40 (0.38, 5.18) | 0.09 |
| Multivariable HR^3^ (95% CI) | 1.00 (ref) | 0.88 (0.33, 2.32) | 1.81 (0.71, 4.58) | 3.84 (1.50, 9.83) | 1.53 (0.37, 6.29) | 0.07 |
| **Folate** | **Q1** | **Q2** | **Q3** | **Q4** | **Q5** | **P _trend_** |
| Median Intake | 11.72 | 12.58 | 13.17 | 13.78 | 14.72 |  |
| Number of Cases | 21 | 11 | 11 | 4 | 8 |  |
| Age-adjusted HR^1^ (95% CI) | 1.00 (ref) | 0.60 (0.29, 1.24) | 0.75 (0.36, 1.55) | 0.37 (0.13, 1.08) | 1.13 (0.50, 2.53) | 0.55 |
| Multivariable HR^2^ (95% CI) | 1.00 (ref) | 0.61 (0.30, 1.27) | 0.78 (0.37, 1.62) | 0.39 (0.13, 1.15) | 1.21 (0.53, 2.78) | 0.79 |
| Multivariable HR^3^ (95% CI) | 1.00 (ref) | 0.57 (0.26, 1.23) | 0.70 (0.30, 1.63) | 0.35 (0.11, 1.17) | 1.01 (0.33, 3.10) | 0.70 |
| **Vitamin E** | **Q1** | **Q2** | **Q3** | **Q4** | **Q5** | **P _trend_** |
| Median Intake | 1.85 | 2.09 | 2.26 | 2.43 | 2.71 |  |
| Number of Cases | 18 | 12 | 15 | 8 | 3 |  |
| Age-adjusted HR^1^ (95% CI) | 1.00 (ref) | 0.78 (0.38, 1.62) | 1.28 (0.65, 2.53) | 0.91 (0.39, 2.09) | 0.43 (0.13, 1.45) | 0.38 |
| Multivariable HR^2^ (95% CI) | 1.00 (ref) | 0.82 (0.39, 1.71) | 1.33 (0.67, 2.68) | 0.95 (0.41, 2.21) | 0.45 (0.13, 1.55) | 0.46 |
| Multivariable HR^3^ (95% CI) | 1.00 (ref) | 0.85 (0.40, 1.81) | 1.39 (0.67, 2.87) | 0.99 (0.41, 2.39) | 0.49 (0.14, 1.77) | 0.59 |
| **Vitamin D** | **Q1** | **Q2** | **Q3** | **Q4** | **Q5** | **P _trend_** |
| Median Intake | 0.58 | 1.14 | 1.51 | 1.89 | 2.46 |  |
| Number of Cases | 15 | 11 | 11 | 12 | 7 |  |
| Age-adjusted HR^1^ (95% CI) | 1.00 (ref) | 0.81 (0.36, 1.80) | 0.70 (0.29, 1.67) | 0.69 (0.27, 1.72) | 0.58 (0.17, 2.12) | 0.27 |
| Multivariable HR^2^ (95% CI) | 1.00 (ref) | 0.79 (0.35, 1.76) | 0.68 (0.29, 1.63) | 0.67 (0.27, 1.68) | 0.49 (0.14, 1.71) | 0.19 |
| Multivariable HR^3^ (95% CI) | 1.00 (ref) | 0.75 (0.33, 1.69) | 0.65 (0.27, 1.55) | 0.62 (0.24, 1.58) | 0.38 (0.10, 1.45) | 0.61 |
| **Magnesium** | **Q1** | **Q2** | **Q3** | **Q4** | **Q5** | **P _trend_** |
| Median Intake | 10.14 | 10.72 | 11.11 | 11.49 | 12.03 |  |
| Number of Cases | 20 | 13 | 9 | 10 | 4 |  |
| Age-adjusted HR^1^ (95% CI) | 1.00 (ref) | 0.82 (0.41, 1.66) | 0.76 (0.35, 1.67) | 1.24 (0.58, 2.66) | 0.92 (0.32, 2.71) | 0.95 |
| Multivariable HR^2^ (95% CI) | 1.00 (ref) | 0.83 (0.41, 1.67) | 0.76 (0.35, 1.69) | 1.24 (0.57, 2.68) | 0.92 (0.31, 2.74) | 0.95 |
| Multivariable HR^3^ (95% CI) | 1.00 (ref) | 0.86 (0.41, 1.77) | 0.81 (0.35, 1.91) | 1.23 (0.50, 3.05) | 1.10 (0.32, 3.77) | 0.98 |
| **Zinc** | **Q1** | **Q2** | **Q3** | **Q4** | **Q5** | **P _trend_** |
| Median Intake | 2.24 | 2.54 | 2.75 | 2.95 | 3.24 |  |
| Number of Cases | 23 | 30 | 17 | 25 | 17 |  |
| Age-adjusted HR^1^ (95% CI) | 1.00 (ref) | 1.08 (0.58, 2.03) | 0.85 (0.39, 1.85) | 1.17 (0.47, 2.89) | 0.80 (0.19, 3.42) | 0.87 |
| Multivariable HR^2^ (95% CI) | 1.00 (ref) | 1.06 (0.56, 1.99) | 0.81 (0.37, 1.78) | 1.11 (0.44, 2.76) | 0.76 (0.18, 3.24) | 0.67 |
| Multivariable HR^3^ (95% CI) | 1.00 (ref) | 1.10 (0.57, 2.14) | 0.85 (0.37, 1.97) | 1.21 (0.45, 3.25) | 1.01 (0.22, 4.79) | 0.84 |

^1^ Adjusted for entry age ^2^Adjusted for entry age, sex (overall), calories, smoking status, race, education, BMI, and physical activity ^3^Additionally adjusted for

vitamin C, vitamin E, beta-carotene, and folate
