# Supplementary material for: The Association between Selenium and Other Micronutrients and Thyroid Cancer Incidence in the NIH-AARP Diet and Health Study
Source: PLoS One. 2014 Oct 20;9(10):e110886. doi: 10.1371/journal.pone.0110886 (PMC4203851; doi:10.1371/journal.pone.0110886)
Supplement: Table S7 — Hazard Ratios (HRs) and corresponding 95% confidence intervals (CIs) for follicular thyroid cancer by quintile of micronutrient intake among men in The NIH-AARP Diet and Health Study. (DOCX) [file pone.0110886.s007.docx]

**Table S7 – Hazard Ratios (HRs) and corresponding 95% confidence intervals (CIs) for follicular thyroid cancer by quintile of micronutrient intake among men in The NIH-AARP Diet and Health Study:**

| **Selenium** | **Q1** | **Q2** | **Q3** | **Q4** | **Q5** | **P _trend_** |
| --- | --- | --- | --- | --- | --- | --- |
| Median Intake | 7.05 | 7.64 | 8.03 | 8.41 | 8.93 |  |
| Number of Cases | 4 | 8 | 8 | 14 | 23 |  |
| Age-adjusted HR^1^ (95% CI) | 1.00 (ref) | 1.11 (0.33, 3.70) | 0.73 (0.22, 2.44) | 1.02 (0.34, 3.09) | 1.48 (0.51, 4.29) | 0.23 |
| Multivariable HR^2^ (95% CI) | 1.00 (ref) | 1.03 (0.31, 3.43) | 0.66 (0.20, 2.20) | 0.91 (0.30, 2.78) | 1.35 (0.46, 3.92) | 0.28 |
| Multivariable HR^3^ (95% CI) | 1.00 (ref) | 1.03 (0.31, 3.46) | 0.66 (0.19, 2.23) | 0.90 (0.29, 2.83) | 1.32 (0.43, 4.03) | 0.33 |
| **Vitamin C** | **Q1** | **Q2** | **Q3** | **Q4** | **Q5** | **P _trend_** |
| Median Intake | 7 | 8.41 | 9.36 | 10.27 | 11.67 |  |
| Number of Cases | 8 | 13 | 11 | 10 | 15 |  |
| Age-adjusted HR^1^ (95% CI) | 1.00 (ref) | 1.59 (0.66, 3.83) | 1.31 (0.53, 3.27) | 1.16 (0.46, 2.93) | 1.61 (0.68, 3.81) | 0.46 |
| Multivariable HR^2^ (95% CI) | 1.00 (ref) | 1.50 (0.62, 3.64) | 1.24 (0.49, 3.10) | 1.08 (0.42, 2.77) | 1.55 (0.64, 3.71) | 0.40 |
| Multivariable HR^3^ (95% CI) | 1.00 (ref) | 1.41 (0.57, 3.49) | 1.13 (0.43, 2.99) | 0.97 (0.34, 2.72) | 1.40 (0.49, 4.02) | 0.75 |
| **Betacarotene** | **Q1** | **Q2** | **Q3** | **Q4** | **Q5** | **P _trend_** |
| Median Intake | 8.67 | 9.38 | 9.89 | 10.43 | 11.3 |  |
| Number of Cases | 7 | 14 | 13 | 10 | 13 |  |
| Age-adjusted HR^1^ (95% CI) | 1.00 (ref) | 1.96 (0.79, 4.85) | 1.85 (0.74, 4.63) | 1.46 (0.56, 3.85) | 2.05 (0.81, 5.16) | 0.27 |
| Multivariable HR^2^ (95% CI) | 1.00 (ref) | 1.88 (0.76, 4.66) | 1.76 (0.70, 4.43) | 1.41 (0.53, 3.73) | 2.01 (0.79, 5.10) | 0.30 |
| Multivariable HR^3^ (95% CI) | 1.00 (ref) | 1.84 (0.73, 4.64) | 1.71 (0.66, 4.47) | 1.37 (0.48, 3.85) | 1.97 (0.69, 5.65) | 0.40 |
| **Calcium** | **Q1** | **Q2** | **Q3** | **Q4** | **Q5** | **P _trend_** |
| Median Intake | 8.67 | 9.38 | 9.89 | 10.43 | 11.3 |  |
| Number of Cases | 7 | 14 | 13 | 10 | 13 |  |
| Age-adjusted HR^1^ (95% CI) | 1.00 (ref) | 1.96 (0.79, 4.85) | 1.85 (0.74, 4.63) | 1.46 (0.56, 3.85) | 2.05 (0.81, 5.16) | 0.27 |
| Multivariable HR^2^ (95% CI) | 1.00 (ref) | 0.68 (0.26, 1.83) | 0.62 (0.23, 1.68) | 0.79 (0.29, 2.16) | 0.84 (0.26, 2.70) | 0.74 |
| Multivariable HR^3^ (95% CI) | 1.00 (ref) | 0.64 (0.24, 1.72) | 0.56 (0.20, 1.56) | 0.70 (0.24, 1.99) | 0.72 (0.21, 2.44) | 0.58 |
| **Folate** | **Q1** | **Q2** | **Q3** | **Q4** | **Q5** | **P _trend_** |
| Median Intake | 11.72 | 12.58 | 13.17 | 13.78 | 14.72 |  |
| Number of Cases | 8 | 7 | 10 | 11 | 21 |  |
| Age-adjusted HR^1^ (95% CI) | 1.00 (ref) | 0.64 (0.23, 1.76) | 0.74 (0.29, 1.88) | 0.68 (0.27, 1.69) | 1.12 (0.50, 2.54) | 0.43 |
| Multivariable HR^2^ (95% CI) | 1.00 (ref) | 0.60 (0.22, 1.66) | 0.70 (0.27, 1.78) | 0.64 (0.25, 1.61) | 1.10 (0.47, 2.54) | 0.43 |
| Multivariable HR^3^ (95% CI) | 1.00 (ref) | 0.54 (0.19, 1.53) | 0.58 (0.21, 1.61) | 0.51 (0.17, 1.46) | 0.85 (0.28, 2.53) | 0.79 |
| **Vitamin E** | **Q1** | **Q2** | **Q3** | **Q4** | **Q5** | **P _trend_** |
| Median Intake | 1.85 | 2.09 | 2.26 | 2.43 | 2.71 |  |
| Number of Cases | 6 | 12 | 14 | 12 | 13 |  |
| Age-adjusted HR^1^ (95% CI) | 1.00 (ref) | 1.56 (0.59, 4.16) | 1.44 (0.55, 3.75) | 1.05 (0.39, 2.79) | 1.03 (0.39, 2.70) | 0.56 |
| Multivariable HR^2^ (95% CI) | 1.00 (ref) | 1.46 (0.55, 3.90) | 1.36 (0.52, 3.54) | 1.00 (0.37, 2.68) | 1.01 (0.38, 2.67) | 0.58 |
| Multivariable HR^3^ (95% CI) | 1.00 (ref) | 1.28 (0.47, 3.45) | 1.11 (0.42, 2.99) | 0.79 (0.28, 2.20) | 0.77 (0.28, 2.17) | 0.58 |
| **Vitamin D** | **Q1** | **Q2** | **Q3** | **Q4** | **Q5** | **P _trend_** |
| Median Intake | 0.58 | 1.14 | 1.51 | 1.89 | 2.46 |  |
| Number of Cases | 5 | 8 | 13 | 20 | 11 |  |
| Age-adjusted HR^1^ (95% CI) | 1.00 (ref) | 1.39 (0.45, 4.31) | 2.09 (0.69, 6.33) | 2.80 (0.90, 8.69) | 1.31 (0.34, 4.98) | 0.43 |
| Multivariable HR^2^ (95% CI) | 1.00 (ref) | 1.32 (0.42, 4.09) | 1.98 (0.66, 5.96) | 2.64 (0.85, 8.15) | 1.26 (0.33 4.80) | 0.46 |
| Multivariable HR^3^ (95% CI) | 1.00 (ref) | 1.30 (0.42, 4.05) | 1.95 (0.64, 5.93) | 2.62 (0.84, 8.22) | 1.28 (0.33, 4.95) | 0.44 |
| **Magnesium** | **Q1** | **Q2** | **Q3** | **Q4** | **Q5** | **P _trend_** |
| Median Intake | 10.14 | 10.72 | 11.11 | 11.49 | 12.03 |  |
| Number of Cases | 3 | 8 | 13 | 17 | 16 |  |
| Age-adjusted HR^1^ (95% CI) | 1.00 (ref) | 1.73 (0.46, 6.54) | 2.16 (0.62, 7.59) | 2.32 (0.68, 7.93) | 1.86 (0.54, 6.40) | 0.42 |
| Multivariable HR^2^ (95% CI) | 1.00 (ref) | 1.65 (0.44, 6.24) | 2.03 (0.58, 7.17) | 2.18 (0.64, 7.50) | 1.79 (0.52, 6.21) | 0.46 |
| Multivariable HR^3^ (95% CI) | 1.00 (ref) | 1.59 (0.42, 6.08) | 1.87 (0.51, 6.86) | 1.92 (0.52, 7.11) | 1.49 (0.37, 5.90) | 0.83 |
| **Zinc** | **Q1** | **Q2** | **Q3** | **Q4** | **Q5** | **P _trend_** |
| Median Intake | 2.24 | 2.54 | 2.75 | 2.95 | 3.24 |  |
| Number of Cases | 2 | 12 | 8 | 19 | 16 |  |
| Age-adjusted HR^1^ (95% CI) | 1.00 (ref) | 3.51 (0.79, 15.72) | 1.53 (0.33, 7.21) | 2.76 (0.64, 11.86) | 2.10 (0.48, 9.14) | 0.84 |
| Multivariable HR^2^ (95% CI) | 1.00 (ref) | 3.17 (0.70, 14.09) | 1.35 (0.29, 6.38) | 2.42 (0.56, 10.48) | 1.87 (0.43, 8.21) | 0.94 |
| Multivariable HR^3^ (95% CI) | 1.00 (ref) | 2.76 (0.60, 12.67) | 1.10 (0.22, 5.44) | 1.83 (0.39, 8.62) | 1.37 (0.28, 6.82) | 0.61 |

^1^ Adjusted for entry age ^2^Adjusted for entry age, sex (overall), calories, smoking status, race, education, BMI, and physical activity ^3^Additionally adjusted for

vitamin C, vitamin E, beta-carotene, and folate
