# Supplementary material for: The Association between Selenium and Other Micronutrients and Thyroid Cancer Incidence in the NIH-AARP Diet and Health Study
Source: PLoS One. 2014 Oct 20;9(10):e110886. doi: 10.1371/journal.pone.0110886 (PMC4203851; doi:10.1371/journal.pone.0110886)
Supplement: Table S8 — Hazard Ratios (HRs) and corresponding 95% confidence intervals (CIs) for follicular thyroid cancer by quintile of micronutrient intake among women in The NIH-AARP Diet and Health Study. (DOCX) [file pone.0110886.s008.docx]

**Table S8 – Hazard Ratios (HRs) and corresponding 95% confidence intervals (CIs) for follicular thyroid cancer by quintile of micronutrient intake among women in The NIH-AARP Diet and Health Study:**

| **Selenium** | **Q1** | **Q2** | **Q3** | **Q4** | **Q5** | **P _trend_** |
| --- | --- | --- | --- | --- | --- | --- |
| Median Intake | 7.05 | 7.64 | 8.03 | 8.41 | 8.93 |  |
| Number of Cases | 21 | 14 | 14 | 5 | 2 |  |
| Age-adjusted HR^1^ (95% CI) | 1.00 (ref) | 0.87 (0.45, 1.72) | 1.38 (0.70, 2.71) | 0.93 (0.35, 2.48) | 0.88 (0.21, 3.76) | 0.82 |
| Multivariable HR^2^ (95% CI) | 1.00 (ref) | 0.91 (0.46, 1.80) | 1.41 (0.71, 2.81) | 0.94 (0.35, 2.51) | 0.86 (0.20, 3.70) | 0.81 |
| Multivariable HR^3^ (95% CI) | 1.00 (ref) | 0.93 (0.46, 1.87) | 1.43 (0.70, 2.93) | 0.95 (0.35, 2.62) | 0.88 (0.20, 3.87) | 0.80 |
| **Vitamin C** | **Q1** | **Q2** | **Q3** | **Q4** | **Q5** | **P _trend_** |
| Median Intake | 7 | 8.41 | 9.36 | 10.27 | 11.67 |  |
| Number of Cases | 15 | 12 | 7 | 9 | 12 |  |
| Age-adjusted HR^1^ (95% CI) | 1.00 (ref) | 0.76 (0.36, 1.63) | 0.44 (0.18, 1.08) | 0.58 (0.25, 1.32) | 0.87 (0.41, 1.87) | 0.75 |
| Multivariable HR^2^ (95% CI) | 1.00 (ref) | 0.76 (0.35, 1.64) | 0.38 (0.14, 0.98) | 0.58 (0.25, 1.35) | 0.89 (0.41, 1.94) | 0.55 |
| Multivariable HR^3^ (95% CI) | 1.00 (ref) | 0.74 (0.34, 1.64) | 0.37 (0.14, 1.01) | 0.59 (0.23, 1.52) | 0.96 (0.35, 2.64) | 0.69 |
| **Betacarotene** | **Q1** | **Q2** | **Q3** | **Q4** | **Q5** | **P _trend_** |
| Median Intake | 8.67 | 9.38 | 9.89 | 10.43 | 11.3 |  |
| Number of Cases | 7 | 15 | 9 | 12 | 13 |  |
| Age-adjusted HR^1^ (95% CI) | 1.00 (ref) | 2.05 (0.84, 5.03) | 1.15 (0.43, 3.09) | 1.43 (0.56, 3.64) | 1.40 (0.56, 3.51) | 0.87 |
| Multivariable HR^2^ (95% CI) | 1.00 (ref) | 2.12 (0.86, 5.21) | 1.20 (0.45, 3.26) | 1.51 (0.59, 3.88) | 1.52 (0.59, 3.88) | 0.81 |
| Multivariable HR^3^ (95% CI) | 1.00 (ref) | 2.23 (0.90, 5.55) | 1.19 (0.42, 3.38) | 1.77 (0.65, 4.80) | 1.99 (0.69, 5.74) | 0.48 |
| **Calcium** | **Q1** | **Q2** | **Q3** | **Q4** | **Q5** | **P _trend_** |
| Median Intake | 8.67 | 9.38 | 9.89 | 10.43 | 11.3 |  |
| Number of Cases | 7 | 15 | 9 | 12 | 13 |  |
| Age-adjusted HR^1^ (95% CI) | 1.00 (ref) | 2.05 (0.84, 5.03) | 1.15 (0.43, 3.09) | 1.43 (0.56, 3.64) | 1.40 (0.56, 3.51) | 0.87 |
| Multivariable HR^2^ (95% CI) | 1.00 (ref) | 0.81 (0.31, 2.13) | 1.61 (0.65, 3.96) | 3.25 (1.33, 7.94) | 1.40 (0.38, 5.18) | 0.09 |
| Multivariable HR^3^ (95% CI) | 1.00 (ref) | 0.88 (0.33, 2.32) | 1.81 (0.71, 4.58) | 3.84 (1.50, 9.83) | 1.53 (0.37, 6.29) | 0.07 |
| **Folate** | **Q1** | **Q2** | **Q3** | **Q4** | **Q5** | **P _trend_** |
| Median Intake | 11.72 | 12.58 | 13.17 | 13.78 | 14.72 |  |
| Number of Cases | 21 | 11 | 11 | 4 | 8 |  |
| Age-adjusted HR^1^ (95% CI) | 1.00 (ref) | 0.60 (0.29, 1.24) | 0.75 (0.36, 1.55) | 0.37 (0.13, 1.08) | 1.13 (0.50, 2.53) | 0.55 |
| Multivariable HR^2^ (95% CI) | 1.00 (ref) | 0.61 (0.30, 1.27) | 0.78 (0.37, 1.62) | 0.39 (0.13, 1.15) | 1.21 (0.53, 2.78) | 0.79 |
| Multivariable HR^3^ (95% CI) | 1.00 (ref) | 0.57 (0.26, 1.23) | 0.70 (0.30, 1.63) | 0.35 (0.11, 1.17) | 1.01 (0.33, 3.10) | 0.70 |
| **Vitamin E** | **Q1** | **Q2** | **Q3** | **Q4** | **Q5** | **P _trend_** |
| Median Intake | 1.85 | 2.09 | 2.26 | 2.43 | 2.71 |  |
| Number of Cases | 18 | 12 | 15 | 8 | 3 |  |
| Age-adjusted HR^1^ (95% CI) | 1.00 (ref) | 0.78 (0.38, 1.62) | 1.28 (0.65, 2.53) | 0.91 (0.39, 2.09) | 0.43 (0.13, 1.45) | 0.38 |
| Multivariable HR^2^ (95% CI) | 1.00 (ref) | 0.82 (0.39, 1.71) | 1.33 (0.67, 2.68) | 0.95 (0.41, 2.21) | 0.45 (0.13, 1.55) | 0.46 |
| Multivariable HR^3^ (95% CI) | 1.00 (ref) | 0.85 (0.40, 1.81) | 1.39 (0.67, 2.87) | 0.99 (0.41, 2.39) | 0.49 (0.14, 1.77) | 0.59 |
| **Vitamin D** | **Q1** | **Q2** | **Q3** | **Q4** | **Q5** | **P _trend_** |
| Median Intake | 0.58 | 1.14 | 1.51 | 1.89 | 2.46 |  |
| Number of Cases | 15 | 11 | 11 | 12 | 7 |  |
| Age-adjusted HR^1^ (95% CI) | 1.00 (ref) | 0.81 (0.36, 1.80) | 0.70 (0.29, 1.67) | 0.69 (0.27, 1.72) | 0.58 (0.17, 2.12) | 0.27 |
| Multivariable HR^2^ (95% CI) | 1.00 (ref) | 0.79 (0.35, 1.76) | 0.68 (0.29, 1.63) | 0.67 (0.27, 1.68) | 0.49 (0.14, 1.71) | 0.19 |
| Multivariable HR^3^ (95% CI) | 1.00 (ref) | 0.75 (0.33, 1.69) | 0.65 (0.27, 1.55) | 0.62 (0.24, 1.58) | 0.38 (0.10, 1.45) | 0.61 |
| **Magnesium** | **Q1** | **Q2** | **Q3** | **Q4** | **Q5** | **P _trend_** |
| Median Intake | 10.14 | 10.72 | 11.11 | 11.49 | 12.03 |  |
| Number of Cases | 20 | 13 | 9 | 10 | 4 |  |
| Age-adjusted HR^1^ (95% CI) | 1.00 (ref) | 0.82 (0.41, 1.66) | 0.76 (0.35, 1.67) | 1.24 (0.58, 2.66) | 0.92 (0.32, 2.71) | 0.95 |
| Multivariable HR^2^ (95% CI) | 1.00 (ref) | 0.83 (0.41, 1.67) | 0.76 (0.35, 1.69) | 1.24 (0.57, 2.68) | 0.92 (0.31, 2.74) | 0.95 |
| Multivariable HR^3^ (95% CI) | 1.00 (ref) | 0.86 (0.41, 1.77) | 0.81 (0.35, 1.91) | 1.23 (0.50, 3.05) | 1.10 (0.32, 3.77) | 0.98 |
| **Zinc** | **Q1** | **Q2** | **Q3** | **Q4** | **Q5** | **P _trend_** |
| Median Intake | 2.24 | 2.54 | 2.75 | 2.95 | 3.24 |  |
| Number of Cases | 23 | 30 | 17 | 25 | 17 |  |
| Age-adjusted HR^1^ (95% CI) | 1.00 (ref) | 1.08 (0.58, 2.03) | 0.85 (0.39, 1.85) | 1.17 (0.47, 2.89) | 0.80 (0.19, 3.42) | 0.87 |
| Multivariable HR^2^ (95% CI) | 1.00 (ref) | 1.06 (0.56, 1.99) | 0.81 (0.37, 1.78) | 1.11 (0.44, 2.76) | 0.76 (0.18, 3.24) | 0.67 |
| Multivariable HR^3^ (95% CI) | 1.00 (ref) | 1.10 (0.57, 2.14) | 0.85 (0.37, 1.97) | 1.21 (0.45, 3.25) | 1.01 (0.22, 4.79) | 0.84 |

^1^ Adjusted for entry age ^2^Adjusted for entry age, sex (overall), calories, smoking status, race, education, BMI, and physical activity ^3^Additionally adjusted for

vitamin C, vitamin E, beta-carotene, and folate
